# The combined detection of Amphiregulin, Cyclin A1 and DDX20/Gemin3 expression predicts aggressive forms of oral squamous cell carcinoma

**DOI:** 10.1038/s41416-021-01491-x

**Published:** 2021-07-21

**Authors:** Ekaterina Bourova-Flin, Samira Derakhshan, Afsaneh Goudarzi, Tao Wang, Anne-Laure Vitte, Florent Chuffart, Saadi Khochbin, Sophie Rousseaux, Pouyan Aminishakib

**Affiliations:** 1 CNRS UMR 5309/INSERM U1209/University Grenoble-Alpes/Institute for Advanced Biosciences, La Tronche, France; 2 Oral and Maxillofacial Pathology Department, School of Dentistry, Tehran University of Medical Sciences, Tehran, Iran; 3 Department of Clinical Biochemistry, School of Medicine, Shahid Beheshti University of Medical Sciences, Tehran, Iran; 4 Cancer Institute Hospital, IKHC, Tehran University of Medical Sciences, Tehran, Iran

**Keywords:** Cancer, Biomarkers

## Abstract

**Background:**

Large-scale genetic and epigenetic deregulations enable cancer cells to ectopically activate tissue-specific expression programmes. A specifically designed strategy was applied to oral squamous cell carcinomas (OSCC) in order to detect ectopic gene activations and develop a prognostic stratification test.

**Methods:**

A dedicated original prognosis biomarker discovery approach was implemented using genome-wide transcriptomic data of OSCC, including training and validation cohorts. Abnormal expressions of silent genes were systematically detected, correlated with survival probabilities and evaluated as predictive biomarkers. The resulting stratification test was confirmed in an independent cohort using immunohistochemistry.

**Results:**

A specific gene expression signature, including a combination of three genes, *AREG, CCNA1* and *DDX20*, was found associated with high-risk OSCC in univariate and multivariate analyses. It was translated into an immunohistochemistry-based test, which successfully stratified patients of our own independent cohort.

**Discussion:**

The exploration of the whole gene expression profile characterising aggressive OSCC tumours highlights their enhanced proliferative and poorly differentiated intrinsic nature. Experimental targeting of *CCNA1* in OSCC cells is associated with a shift of transcriptomic signature towards the less aggressive form of OSCC, suggesting that CCNA1 could be a good target for therapeutic approaches.

## Introduction

Oral squamous cell carcinoma (OSCC) is associated with high morbidity and mortality rates, despite significant progress in therapeutic strategies and modalities [1, 2].

Although the histopathological scaling system [3] is regarded as a practical and easily usable, standard approach to cancer prognostic, today there is still a need for markers which would efficiently predict outcome and guide clinicians towards more precise and personalised therapeutic approaches.

In a recent review and meta-analysis of the literature, Rivera et al. [4]. who identified 41 proteins candidates as prognosis markers in OSCC, mostly detected by immunohistochemistry (IHC), also demonstrated that these candidate markers lack confirmation and required validation before being proposed for clinical use. A similar conclusion was reached by authors who carried out a thorough meta-analysis of candidate prognosis biomarkers focused on oral tongue squamous cell carcinoma [5, 6].

Various molecular and cellular factors have also been proposed to help the prognosis for OSCC patients. For instance, the quantification of tumour-associated infiltrating immune cells [7, 8] or the detection of HPV. Indeed, HPV-positive OSCC is associated with longer survival and more favourable outcomes than HPV-negative tumours [9].

However, the discovery and validation of prognostic markers, which could be detected by routine laboratory techniques, including immunostaining, is still in need.

The use of concept-driven approaches to explore tumour heterogeneity is an efficient way towards the identification of such markers. The strategy we propose here is to use the cancer-specific normally silent gene activation as a lead to biomarker discovery.

Indeed, the occurrence and development of cancers are associated with large-scale genetic abnormalities and epigenetic disorganisations leading to major deregulations in gene expression programmes, and therefore abnormal gene repressions and activations. In particular, cancer cells express genes whose normal expression patterns are restricted to or largely predominant in one specific organ or stage of development, and which are normally epigenetically repressed in most tissues and cell types. Remarkably, although mostly overlooked for many years, abnormal gene expressions have recently been revealed as major actors in cancer development and aggressiveness (for review, see refs. [10–15]).

A systematic search for abnormally expressed genes, by mining expression data of several thousands of cancers of various origins, revealed that they actually occur in all cancers [16] and their detection in various malignancies, including lung cancer [16, 17] and haematological malignancies [18, 19], demonstrated that they provide a very interesting source of new cancer biomarkers and potential targets for new therapeutic approaches [17, 18, 20].

Here, a similar approach was applied to detect the abnormal expression of tissue-predominant genes in OSCC and to test the association between the gene activations and survival probabilities. The approach was actually adapted so that RNAseq data, which have become widely available today, could be exploited in the identification of genes with a tissue-specific or predominant expression as well as in the use of cancer RNAseq data (available on TCGA).

Hence, by mining transcriptomic microarray and RNAseq data from two available independent series of OSCC tumours, respectively used for training and validation, abnormal gene expressions were identified, which were significantly associated with shorter survival in patients. In particular, a subset of three genes, namely *AREG, CCNA1* and *DDX20*, was selected because of its efficiency in prognostic stratification of OSCC patients and the availability of antibodies against the proteins they encode. This combination of three genes was further validated in the large TCGA dataset and in multivariate analyses with other prognostic clinical and biological parameters. A prognosis stratification test based on the IHC detection of the three proteins was conceived and successfully applied to our own independent cohort of patients. Furthermore, by characterising the genome-wide expression signature associated with the 3-genes-positive aggressive tumours and comparing it with that of a cell line where CCNA1, one of the three genes, had been down-regulated, we observed a shift of this transcriptomic signature towards that of the less aggressive form of OSCC, suggesting that CCNA1 could be a good target for therapeutic approaches.

## Methods

In a search for new prognostic markers in OSCC, we applied a data-mining approach that we had previously developed and used for the identification of cancer biomarkers in lung cancer [16, 17], B acute lymphoblastic leukaemia (ALL) [19] and lymphomas [18].

### Rationale

The initial exploration step aims at identifying, among genes abnormally expressed in cancer, strong candidates with high potential for the encoded proteins to be used as a basis for prognosis IHC tests. Here, candidate markers are first identified using gene expression data, which are available on a genome-wide basis in cohorts of tumour samples. However, in most cases, expression levels measured by mRNA quantification are highly unlikely to be correlated with the semi-quantitative measurement of IHC antigenic signals of the encoded protein. Therefore, most candidate markers identified using gene expression data may not be giving informative results when used in IHC. In order to increase our chances to identify relevant candidates for developing IHC tests, we therefore focussed on genes whose expression is normally absent in non-tumour tissue, meaning genes that normally have a specific or highly predominant expression in tissues other than adult somatic tissues. Another requirement is to identify genes whose expression level not only is associated with survival probability but also where a threshold can be defined with enough stability so that different technologies are likely to give similar outcomes in terms of association with survival.

### Study design

The marker discovery approach is based on an original approach that we specifically designed to exploit publicly available transcriptomic data in cancer in order to identify aberrantly expressed genes as new candidate prognostic biomarker genes in oral squamous cell carcinoma (OSCC), which were then combined into a prognostic stratification test, and further validated in a second independent cohort of patients with transcriptomic data. An IHC detection test was designed on the basis of this gene combination and validated in our patients, a third retrospective cohort of patients with a primary OSCC tumour, diagnosed between 1994 and 2015, whose follow-up ranged from 3 to 262 months with a median at 193 months (see Supplementary Methods). Written consents were obtained from all patients for their data/specimens to be used for research purposes. The REMARK guidelines for marker discovery were applied [21].

In all three cohorts of patients, the main clinical endpoint examined was overall survival, which was correlated with the expression of the biomarker genes (microarray or RNAseq transcriptomic data, 1st and 2nd cohorts) or proteins (IHC, 3rd cohort). Age, gender, tumour anatomic site, tumour grade (I, II and III, respectively, corresponding well, moderately and poorly differentiated tumours), TNM stages as well as HPV status were also considered and included in models when available. For our cohort (3rd), the evaluation of sample size is detailed in the Supplementary Method.

### Main steps of our marker discovery approach

**Fig. 1:**
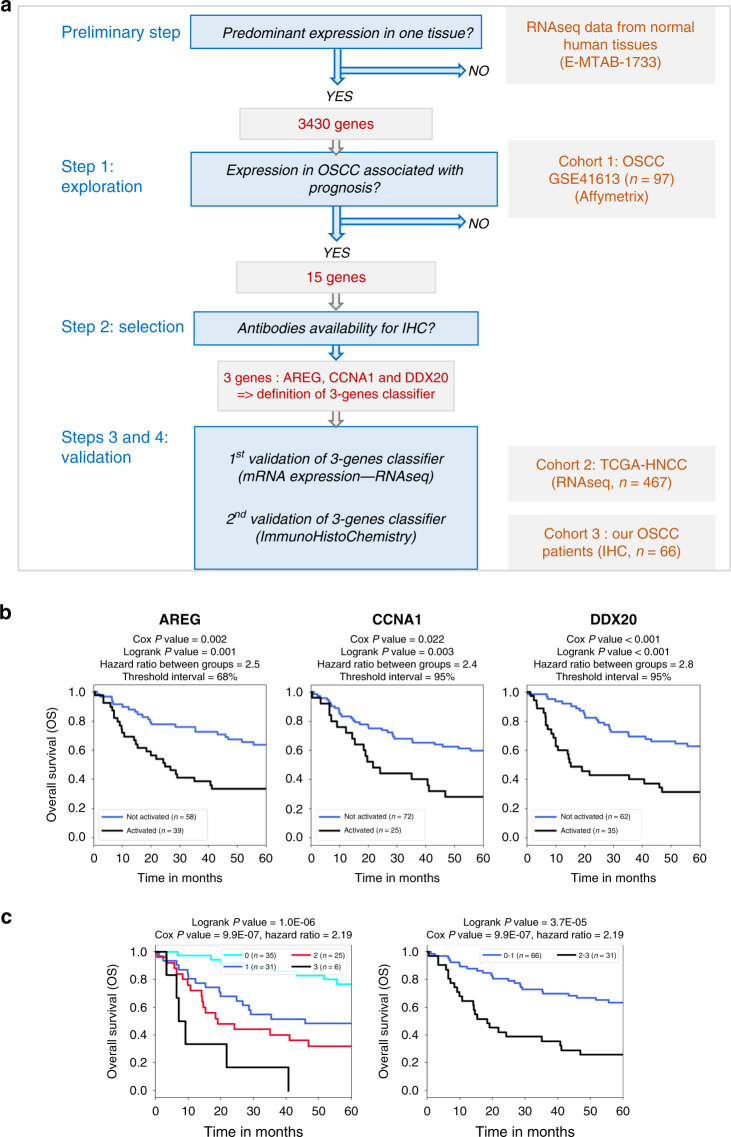
Identification of candidate prognostic biomarkers in OSCC. **a** Overview of the strategy to identify prognostic biomarkers in OSCC (oral squamous cells carcinoma). From the analysis of RNAseq data publicly available from normal human tissues (Arrayexpress: https://www.ebi.ac.uk/arrayexpress/experiments/E-MTAB-1733/), 3430 candidate genes were identified with a predominant expression in one specific tissue-type, and either silent or with a very low expression level in all normal non-germline adult tissues. Step 1: exploration: By analysing the expression of these genes in transcriptomic data of the first series of 97 oral squamous cell carcinoma (OSCC) tumours with survival data (https://www.ncbi.nlm.nih.gov/geo/query/acc.cgi?acc=GSE41613), 15 genes were selected as prognostic marker candidates according to three criteria: (1) significant association between overexpression and a shorter survival probability (Cox model *P* value < 0.05), (2) existence of expression thresholds stratifying patients with significantly different survival probabilities (log-rank test *P* value < 0.05), (3) interval of significant thresholds >50% (in percentiles). Step 2: selection: Of the 15 genes satisfying these three criteria, three were chosen, on the basis of the availability of a reliable antibody able to specifically detect the corresponding proteins. Step 3: 1st validation of the 3-genes combination: The efficiency of their combination to stratify patients with different prognoses was validated in an independent cohort constituted of the 467 HNSC (head and neck squamous carcinoma) patients from the TCGA (from https://portal.gdc.cancer.gov/projects/TCGA-HNSC) in univariate as well as multivariate analyses taking into account clinical and biological parameters associated with prognosis in OSCC. Step 4: 2nd validation of the 3-genes combination by immunohistochemistry (IHC): This 3-genes-based classifier was used to define an IHC test based on the detection of the corresponding proteins, which successfully predicted survival in our series of 66 patients. **b**, **c** Stratification of the OSCC patients using the 3-genes signature in the training dataset (GSE41613, *n* = 97, Affymetrix technology). **b** Individual ectopic expression of each gene: Kaplan–Meier survival curves illustrating the association between the abnormal expression of each of the three selected genes (*AREG, CCNA1*, and *DDX20*) and overall survival probability in OSCC patients. For each gene, the survival probabilities are compared between OSCC patients whose tumour had activated the gene (black line) and those whose tumour had not activated the gene (blue line). The *P* values of the Cox and log-rank models are shown. **c** Combination of three genes by the number of ectopically expressed genes: Kaplan–Meier survival curves comparing the overall survival (OS) probability between OSCC patients grouped according to the number of activations of the combination of the three selected genes (3-genes encompassing *AREG, CCNA1* and *DDX20*). In the left panel, the patients are grouped according to the total number of gene activations (as indicated), whereas in the right panel the survival is compared between two groups of patients whose tumour activates none or only one of the three genes (blue curve) or two or three of the three genes (black curve). The number of activated genes is used as the explanatory variable in the cox model, whereas the log-rank tests the significance of a different survival probability between groups.

An overview of the strategy is presented in Fig. 1a and its legend. A detailed description of the datasets and the approach is provided in the Supplementary Method section.

#### (1) Identification of tissue-predominant genes abnormally expressed in OSCC and whose expression is associated with shorter survival

Our biomarker discovery strategy was updated to adapt it to the use of RNAseq data as a source of gene expression measurement in normal tissues as well as in cancers. By exploiting publicly available RNAseq data in normal tissues (GTEx and E-MTAB-1733 public databases), we first identified 3430 genes predominantly expressed in one human tissue, and not expressed or expressed only at low levels in most adult somatic (non-germline) tissues. This situation enabled us to establish threshold values of transcriptomic signals, which could distinguish between expression activation and none or low (background) expression in cancer.

Hence, this list of genes served as a basis for the detection of abnormal gene expressions in OSCC tumours.

The first step of our approach was an exploration step aiming at the identification of genes whose aberrant expression is significantly associated with shorter survival in OSCC. The Affymetrix dataset GSE41613 with available survival data was used as a training cohort and three criteria were applied for gene selection, which included: (1) a significant association between the level of expression and survival probability (Cox model *P* value < 0.05), (2) the existence of a range of thresholds defining two groups of patients with significantly different survival probabilities (log-rank *P* values < 0.05), (3) an interval of significant thresholds >50% (see Fig. 1a, step 1 and the corresponding legend). The list of 15 genes selected according to these criteria and the corresponding univariate analysis outputs are shown in Supplementary Tables S1 and S2A.

#### (2) Design of a three-genes based prognostic stratifying system and first validation using an independent cohort with transcriptomic data

The 2nd step of our approach consisted of the selection of genes to design a test that could be used in combination to stratify patients.

Three genes, respectively, encoding Amphiregulin (AREG), Cyclin A1 (CCNA1) and DEAD (Asp–Glu–Ala–Asp) box polypeptide 20 (DDX20), were selected to design a stratifying system using their combination.

The 3rd step consisted in the validation of the association between the expression of these three genes and prognosis in an independent cohort of patients with transcriptomic data from the TCGA-HNSC dataset (467 patients with RNAseq and survival data).

#### (3) Stratification of the OSCC patients according to the protein/antigenic signature using IHC on tumour sections in our cohort of OSCC patients

The last step of our approach was the 2nd validation of our prognostic test in our cohort of OSCC patients.

In order to finally propose a prognostic test that could be implemented by pathologists at the time of diagnosis, we decided to set up and use an IHC test for the detection of the corresponding three proteins on tumour sections. We based our choice of antibodies on the literature, and the chosen antibodies are described in Supplementary Table S3. The specificities of the three antibodies were checked on western blots using extracts from three frozen OSCC tumour samples and two non-tumour samples from adjacent tissues.

This IHC test was applied on tumour sections from our cohort of 66 OSCC patients for whom survival data were available.

### Patients and biological samples

Paraffin-embedded samples of 66 patients with OSCC were retrospectively collected from two Pathology laboratories (School of Dentistry, Cancer Institute Hospital) of the Tehran University of Medical Sciences (TUMS). Patients included for this study had primary OSCC tumour which had not received chemo or radiotherapy prior to tumour resection and had follow-up data ranging from 3 to 262 months, with a median at 193 months and 14 events out of 66 patients. Patients with a history of other malignancy and patients with death unrelated to OSCC were excluded from the study. Written consents were available from all patients for their data/specimens to be used for research purposes.

The clinical and pathological data associated with these samples, as well as the patients’ treatments and follow-up data are detailed in Supplementary Table S4A and summarised in Supplementary Table S4B. Specimens were obtained from incisional biopsies or total excised lesions. All OSCC histology slides previously stained with haematoxylin–eosin (HE) were reviewed by two pathologists to confirm the diagnosis. The specimens were graded as follows: well (Grade I), moderately (Grade II) or poorly differentiated (Grade III) according to the WHO criteria. Clinical data such as gender, tumour location, size of the primary tumour (T) and, when available, regional lymph node metastasis (N) and distant metastasis (M) were also collected from the patient’s information file. Tumour staging was performed according to the AJCC guidelines. Follow-up time and survival status (dead or alive) were also recorded. All deaths from this cohort were due to cancer or cancer-related causes.

This study was approved by the Research Ethical Committee of Tehran University of Medical Sciences (Ethical Code: IR.TUMS.DENTISTRY.REC.1397.001: April 29, 2018), and all specimens’ processing and patients’ data gathering were done under the supervision of this committee.

Tumours were resected according to the standardised and approved treatment plan for OSCC patients. The specimens were transferred to the Pathology laboratory in 10% buffered formalin fixative solution and were processed to paraffin blocks, which were stored in the archives of the Pathology laboratory.

### Immunohistochemistry assay

All paraffin blocks were cut into 4-µm slices by a microtome (Rotary microtome, DS-8402, IRAN) and the sections were mounted on glass slides previously immersed in 50% ethanol 96%/water solution and warm water/gelatin, respectively. The slides were then incubated at 38–40 °C for 24 h. The immunoperoxidase method was used for immunodetection. The standardisation of immunohistochemical reactions was done using paraffin-embedded control human tissue, including testis (for CCNA1 and AREG) and breast (for DDX20), based on known immunoreactivity of the corresponding antibodies [22–24]. For negative controls, the primary antibody was omitted and replaced with PBS (phosphate-buffered saline).

The sections were immersed in xylene for deparaffinization and rehydrated in ethanol, then incubated in PBS 3% hydrogen peroxide for 30 min. Antigen retrieval was then performed by immersion in 10 mM citrate buffer (pH 6.0) in a steamer for 15 min at 90 °C followed by a 10-min incubation in Protein Block Serum-Free (Dako, Carpinteria, CA) to block nonspecific reactions. The sections were then incubated with the primary antibodies for 18 h at 41 °C, washed twice with PBS (pH 7.4) and then incubated with the secondary antibody (using the appropriate detection system) for 30 min, then washed in PBS. After dehydration, they were mounted in Permount resin and observed under a light microscope.

### Analysis of immunostained samples

The analysis of the immunostained samples was performed blindly by two pathologists with expertise in oral and maxillofacial histology, who were not aware of the clinicopathological findings. Slides with immunodetection of each of the three marker proteins were successively examined for each patient with Olympus X51 light microscope (Japan). Positive immunoreactivity was defined by tumour cells exhibiting brown staining in the nucleus (for AREG) or cytoplasm (for CCNA1 and DDX20). It is of note that in our OSCC series, CCNA1 was predominantly cytoplasmic. This subcellular localisation was different from what had been observed previously in HNSCC by Weiss et al. [25], who describe a predominantly nuclear localisation in tumour samples. However, other published works clearly show that the subcellular localisation of CCNA1 is context-dependent, with a nuclear localisation in non-tumour cells, i.e., male germ cells or normal hematopoietic cells, but predominantly cytoplasmic in malignant cells, such as ovary tumours [26] or leukemic cells [27].

For each marker, the slides were scored according to the proportion of positive tumour cells and staining intensity. The thresholds between “high” and “low” expressions were adjusted as follows for each of the three antibodies. For the AREG antibody, cases with >50% positive tumour cells were considered as “high”. For the CCNA1 antibody, the staining intensity was scored from 0 (no staining) to 3 (very intense staining), another score was given according to the proportion of stained cells of 1 or 2 if less or more than 10% of tumour cells were stained, respectively, then the intensity score and the proportion score were multiplied, and a case was considered “high” if the intensity ×  proportion score was 3 or above (meaning level 3 intensity score or more than 10% of positive cells). For the DDX20 antibody, the staining intensity was scored from 0 (no staining) to 3 (very intense staining), the proportion scores were 1, 2 or 3 if the proportion of positive tumour cells were <33%, 34–66% or >66%, respectively, and a case was considered “high” if the intensity x proportion score was 3 or above.

#### (4) CCNA1 inhibition in the OSCC cell line FaDu (ATCC), FACS, RNAseq data generation and differential expression analysis

These steps are detailed in Supplementary Methods.

#### (5) Molecular characterisation of aggressive forms of OSCC

In order to explore the transcriptomic profiling of the OSCC tumours expressing at least two of the three genes of our gene expression classifier (3-genes positive) and compare it to the other tumours (expressing none or only one of the three selected genes, 3-genes negative), a differential analysis of transcriptomes between 3-genes positive and negative tumours was performed followed by a Genesets Enrichment Analysis approach, as detailed in Supplementary Methods (GSEA: http://www.gsea-msigdb.org/gsea/index.jsp, using MsigDB datasets: http://www.gsea-msigdb.org/gsea/msigdb/search.jsp).

## Results

The main steps of the study are represented in Fig. 1a.

### (1) Identification of 15 genes whose expression is significantly associated with shorter survival in OSCC

Our first step consisted in identifying genes whose expression is significantly associated with shorter survival in the 97 OSCC patients of the Affymetrix dataset GSE41613 with available survival data (Fig. 1a, step 1). The approach is detailed in Supplementary Fig. S1 and its legend.

A total of 15 genes were found significantly associated with shorter survival probability in a univariate analysis and satisfying these criteria (Supplementary Fig. S2 and Supplementary Tables S1 and S2).

These genes were all protein-encoding genes (Supplementary Table S1). They were encoding for amphiregulin (AREG), cyclin A1 (CCNA1), cyclin-dependent kinase inhibitor 3 (CDKN3), DEAD-box helicase 20 (DDX20), EF-hand calcium-binding domain 11 (EFCAB11), guanine monophosphate synthase (GMPS), matrix metallopeptidase 10 (MMP10), MRG domain-binding protein (MRGBP), nucleoporin 155 (NUP155), pappalysin 1 (PAPPA), PSMC3 interacting protein (PSMC3IP), proteasome assembly chaperone 1 (PSMG1), suppressor of variegation 3–9 homologue 2 (SUV39H2), tryptophan 2,3-dioxygenase (TDO2), thyroid hormone receptor interactor 13 (TRIP13).

Our aim was to identify markers, which would be readily available for pathologists to help establishing a prognosis in OSCC patients and optimise therapeutic strategies accordingly. IHC is a technique that can be performed on tumour sections by pathologists, but its performance is highly dependent on the availability of antibodies, with high sensitivity and specificity.

Considering the list of the 15 candidate genes, three genes were identified, respectively, encoding Amphiregulin (AREG), Cyclin A1 (CCNA1) and DEAD (Asp–Glu–Ala–Asp) box polypeptide 20 (DDX20), for which commercially available antibodies suitable for immune staining could be found.

The association between the expression of each of these three genes and the prognosis of the OSCC patients of this training cohort (dataset GSE41613) is illustrated in Fig. 1b. In order to test the ability of these three genes in combination to predict prognosis more accurately than each individually, the 97 patients of the training cohort were also grouped according to the sum of positive expressions (0, 1, 2 or 3) and survival probabilities were compared between the groups of patients (Fig. 1c, left panel). In particular, groups of comparable sizes showing a highly significant difference in survival probabilities were obtained by stratifying the patients into two groups: 0 or 1 activated genes corresponded to the longest survival probability (“good” prognostic) whereas 2- or 3-gene activations predicted shorter survival probability (“poor” prognostic) (Fig. 1c, right panel). This cohort included patients with different tumour stages associated with different survival probabilities (Supplementary Fig. S3). Multivariate analyses including gender, stage and age as well as our 3-gene classifying system (3-genes) demonstrated that both stage and 3-genes were strong predictors of prognosis (Supplementary Table S5).

### (2) The abnormal expression of a combination of three genes is consistently associated with shorter survival probability in an independent cohort of HNSC patients

**Fig. 2:**
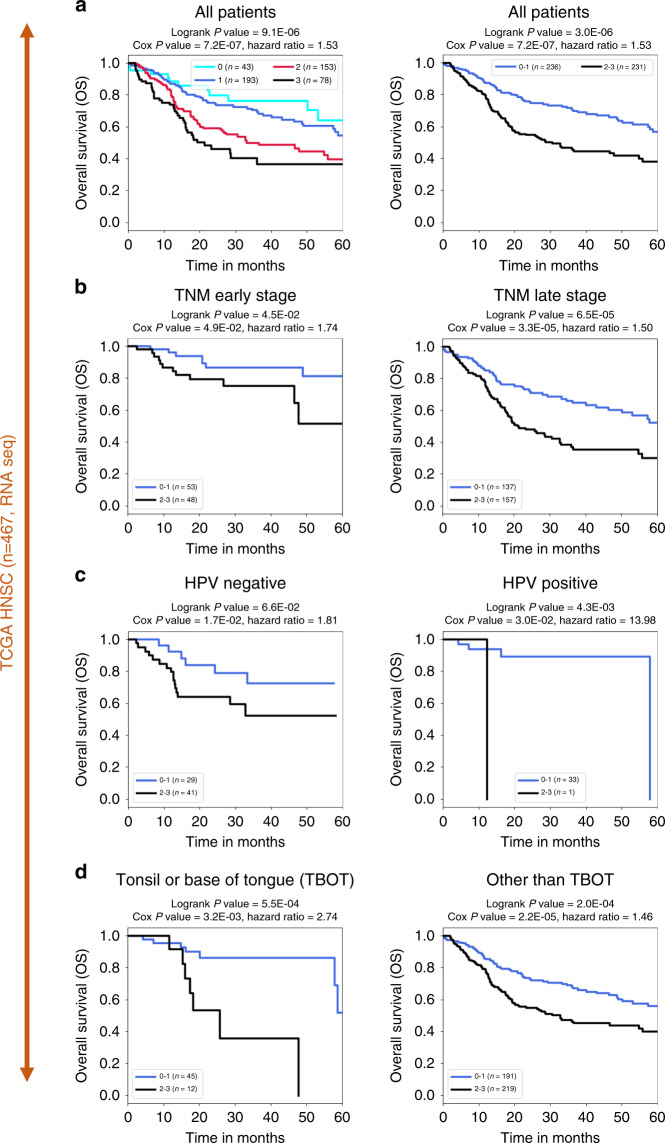
Stratification of the TCGA-HNSC patients using the 3-genes signature in the validation dataset (*n* = 467, RNAseq). Kaplan–Meier survival curves illustrating TCGA-HNSC patients’ overall survival (OS) according to the number of activations of the three genes (*AREG, CCNA1* and *DDX20*), considering all patients (*n* = 467) (**a**) or subsets of patients defined according to clinical and biological parameters (**b**, **c** or **d**) including TNM early or late stages (**b**), human papillomavirus (HPV) status (**c**) or tumour localisation (**d**). **a** All patients: in the left panel, the patients are grouped according to the total number of gene activations, and in the right panel survival probabilities are compared between patients with tumours activating none or one gene (0–1) or 2 or 3 of the three genes (2–3). **b**–**d** Kaplan–Meier survival curves illustrating TCGA-HNSC patients’ overall survival (OS) according to our 3-gene classifier when applied to the following subsets of patients. **b** Survival probabilities of subsets of patients at an early TNM stage ((T1 or T2) and (N0 or N1), left panel) or TNM late-stage ((T3 or more) or (N2 or more), right panel). **c** Survival probabilities of subsets of HPV-negative patients (left panel) or HPV-positive patients (right panel, note that only one HPV-positive patient is negative for our 3-gene classifier and *P* values and hazard ratio are not statistically relevant in this case). **d** Survival probabilities of subsets of patients with tonsil or base of tongue localisation (TBOT: left panel) or other localisation (other than TBOT: right panel).

Remarkably, the prognostic power of this 3-gene combination (3-genes) was confirmed using expression data obtained from an independent cohort of patients with a different technology, RNAseq (TCGA-HNSC dataset : 467 patients with survival data). The patients were grouped as above according to the sum of positive activations of these three genes and their survival probabilities were compared, demonstrating the ability of these three genes in combination to predict prognosis in this independent population of patients (Fig. 2a). The three genes were also individually associated with shorter survival in this cohort of patients, although for DDX20 this correlation did not reach our criteria of significance (Supplementary Fig. S4), further supporting the advantage of using several genes in combination.

Since several clinical and biological parameters potentially relevant to establish prognosis were available for this latter cohort (Supplementary Fig. S5), it was possible to compare the prognosis predictive value of our 3-gene combination with that of these other parameters.

Of the bio-clinical parameters that were available for this cohort, TNM stages, HPV status and the anatomic site, were confirmed as prognosis predictors (Supplementary Fig. S5). When applied to subsets of patients, which had already been stratified with TNM stage, HPV status, or anatomic site of the tumour (tonsil or base of the tongue versus other sites), our 3-genes classifier provided an informative and efficient sub-stratification into prognosis groups (Fig. 2b–d).

Interestingly, the 3-gene-based test assigned nearly all HPV-positive OSCC to the “good” prognostic group, whereas it remained highly informative in HPV-negative OSCC since it successfully sub-stratified patients with significantly different prognoses (Fig. 2c). Multivariate survival analyses confirmed that the 3-gene-based classifier is a highly informative prognostic predictor (Supplementary Table S5).

### (3) Stratification according to the protein/antigenic signature using IHC on tumour sections also confirms a highly significant association with poor prognosis in our cohort of OSCC patients

We then developed an IHC test which we applied on tumour sections from our cohort of 66 OSCC patients for whom various clinical records, including survival data, were available, and are detailed in Supplementary Table S4 and Supplementary Fig. S6.

Antibodies specific against the three proteins encoded by the three genes were purchased (Supplementary Table S3), and their respective specificities were checked by western blots using extracts from three frozen OSCC tumour samples and two non-tumour samples from adjacent tissues (Supplementary Fig. S7).

The corresponding IHC test was applied on tumour sections from our cohort of 66 OSCC patients. The detailed IHC results for each patient are shown in Supplementary Table S4A.

**Fig. 3:**
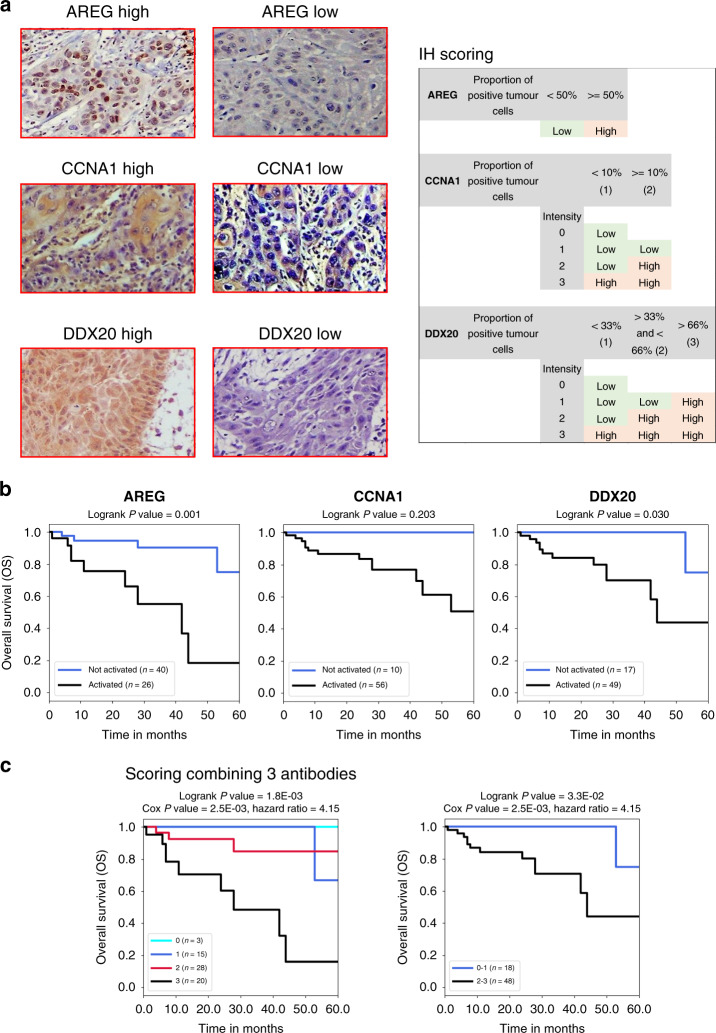
Stratification of the OSCC patients according to the protein/antigenic signature using IHC on tumour sections in our validation cohort of OSCC patients. **a** Left panel: examples of immunohistochemical detection of AREG, CCNA1 and DDX20, as indicated, showing samples with “high” and “low” labelling at an x400 magnification. Right panel: scoring criteria are shown (see the methods section for details). **b** Prognostic of patients grouped according to the IHC detection of each of the three proteins individually: high labelling = 1 and low labelling = 0. **c** Prognostic of patients stratified with the three proteins in combination (the score corresponds to the total of “high” scorings for the three antibodies).

Interestingly, the detection of the proteins encoded by these three genes by IHC in our cohort of OSCC patients (illustrated and described in Fig. 3a and Supplementary Fig. S8), confirmed the association between high expression of each of these proteins and shorter survival, reaching significance for AREG and DDX20 (Fig. 3b).

Remarkably, the IHC profiling of the patients obtained by combining the three antibodies resulted in a patient’s stratification that was also highly associated with prognosis. Patients with no or only one IHC positive expression display longer survival probability than those with two or three positive IHC tests (Fig. 3c).

This result demonstrates not only a successful validation of new biomarkers in an independent cohort of patients using a different technology but also paves the way for the development of a test relatively simple to implement in the context of a pathology lab, which would provide useful complementary information for the clinicians at the time of diagnosis.

### (4) The whole-genome expression profile of aggressive forms of OSCC reveals a specific molecular profile, which could serve as a basis for adapting therapeutic strategies

In addition to the direct clinical purpose of this investigation, the ability to identify aggressive forms of cancer by using this approach also enables an extensive molecular characterisation of the tumours based on their whole-genome transcriptomic profiling.

**Fig. 4:**
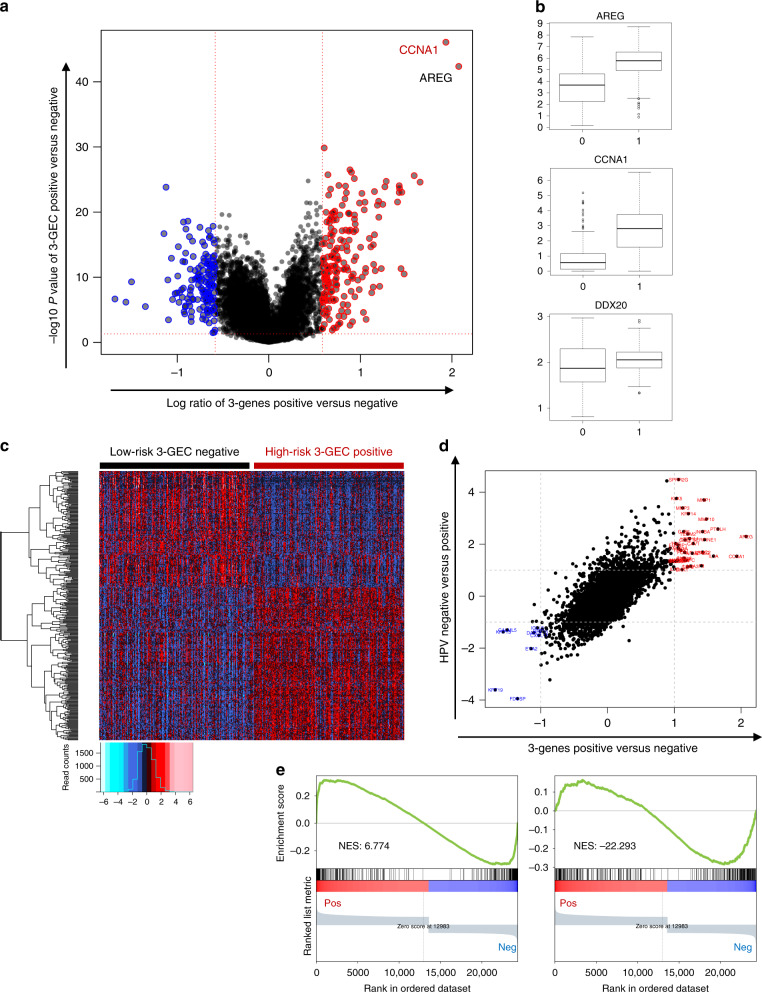
Differential analysis between 3-genes positive and negative OSCC tumours identifies a genome-wide expression signature in dataset TCGA-HNSC. **a** Volcano plot showing log ratio (*x* axis) and −log10 (*P* value) (*y* axis) of differential expression between 3-gene-positive OSCC samples (>= 2 activated genes) and the low-risk 3-genes negative samples (no or one gene activation). **b** Box plots showing the distribution of expression values of the three genes between the two groups of tumours defined above. 0 = low-risk 3-gene-negative samples; 1 = high-risk 3-genes positive samples. **c** Heatmap illustrating the expression of 339 genes up- or downregulated (t-test *P* value < 0.01, absolute fold change value > 2) in the high-risk 3-gene-positive OSCC samples compared with the low-risk 3-gene-negative samples. **d** Correlation plot between the 3-genes positive versus negative signature (*x* axis) and HPV-negative versus positive signature (*y* axis) (correlation coefficient = 0.72). **e** The gene expression profile of HPV-negative versus -positive OSCC tumours shares similarities and differences compared to the signature of 3-genes positive versus negative OSCC tumours in TCGA-HNSC dataset. GSEA plots showing the following genesets ranked according to the log ratio between 3-genes positive versus negative OSCC tumours in the TCGA-HNSC dataset. Left panel: genes depleted in HPV-negative versus positive OSCC tumours (fold change < −2 and *P* value < 0.01) (*n* = 357 genes). A large proportion of these genes are also downregulated in 3-genes positive tumours compared to 3-genes negative. Right panel: genes enriched in HPV-negative versus positive OSCC tumours (fold change > 2 and *P* value < 0.01) (*n* = 393 genes). Many of these genes are also upregulated in 3-genes positive tumours compared to 3-genes negative.

Differential analysis of transcriptomes between 3-genes positive aggressive tumours and the others (Fig. 4a–c), followed by a Gene Set Enrichment Analysis (GSEA) approach, enabled us to have global insight into the biology of these aggressive forms of OSCC.

GSEA plots obtained for a selection of informative genesets from the publicly available genesets of the C2, C5 and h categories of the Msig database (http://software.broadinstitute.org/gsea/msigdb/index.jsp) are shown Supplementary Fig. S9 and genesets enrichment scores and composition are detailed in Supplementary Table S6.

The main characteristics of the expression profiles of aggressive forms of OSCC as detected by the 3-genes classifier are highly similar in both datasets, from GSE41613 and TCGA-HNSC. They include (i) shared signatures with highly cycling and proliferative cells as well as with embryonic stem cells, an observation similar to that of aggressive lung tumours [16], (ii) an upregulation of signatures corresponding to poorly differentiated tumours as well as of genes involved in epithelial mesenchymal transition (EMT) and (iii) an upregulation of genes involved in various oncogenic signatures, including HRAS, TNFA/NFKB and EGF-associated signatures (Supplementary Fig. S9A, B).

### (5) Experimental targeting of *CCNA1* in OSCC cells is associated with a shift of transcriptomic signature towards the less aggressive form of OSCC

Since the expression of *CCNA1* was robustly associated with poor prognosis, and that its encoded protein is a cyclin-dependent kinase that could potentially be targeted by specific drugs, we also experimentally tested the effect of the inactivation of this gene on the proliferative ability and on the transcriptomic profile of OSCC cells.

**Fig. 5:**
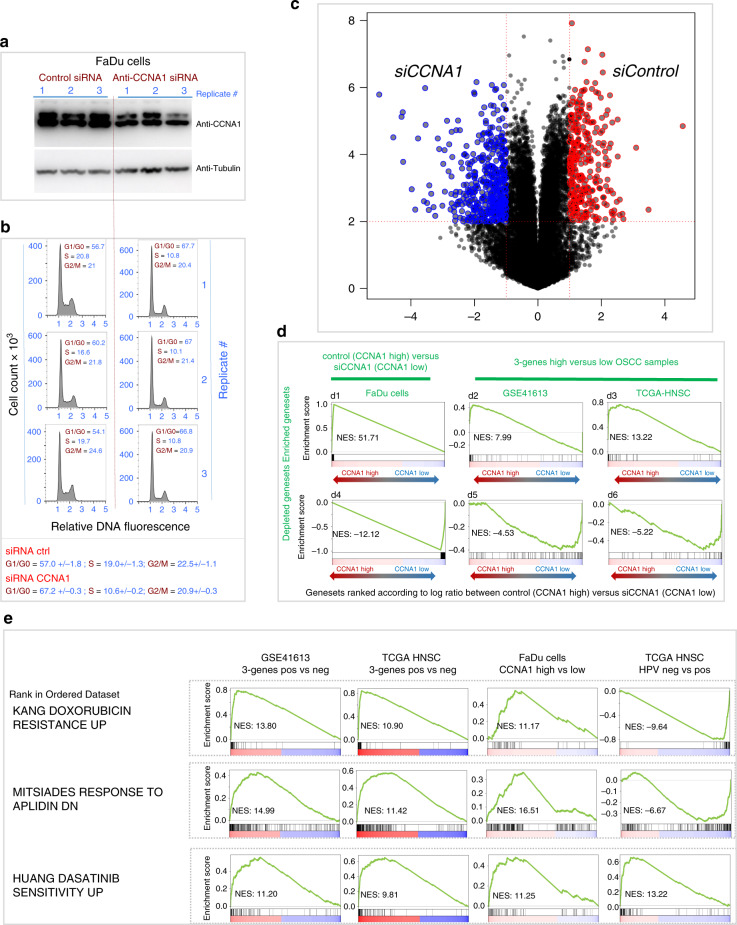
Downregulation of CCNA1 decreases cell proliferation and induces a shift of the gene expression profile toward that of 3-genes negative good prognosis OSCC tumours. **a** CCNA1 knockdown in FaDu OSCC cell line. Western blots showing the decreased expression of the CCNA1 protein in FaDu cells after knockdown of the gene. In all, 20 µg of urea extracts from cells transfected either with siCCNA1 or with SiControl were analysed by immunoblotting using anti-CCNA1 (upper panel) and anti-tubulin (lower panel) antibodies. Three replicates of each condition were done. **b** FACS analysis of cell cycle in FaDu cells after siCCNA1. The cell cycle of FaDu cells transfected either with siCCNA1 or with SiControl was analysed by Flow Cytometry using the Accuri C6 Flow Cytometer. Three replicates of each condition were done. **c** Volcano plot showing the log ratio (*x* axis) and −log10 (*P* value) (*y* axis) of differential expression between control and siCCNA1 FaDu cells. **d** The gene expression profile of control versus siCCNA1 treated FaDu cells is similar to that of 3-genes positive versus negative OSCC tumours in both datasets. GSEA plots showing the following genesets ranked according to the log ratio between control (CCNA1 high) versus siCCNA1 (CCNA1 low) FaDu cells in our RNAseq experiment: d1, d4. Genes enriched or depleted in CCNA1 high versus low FaDu cells with absolute fold change > 2 and *P* value < 0.01 in control versus siCCNA1 FaDu cells) (respectively, *n* = 314 genes or *n* = 482 genes) (to illustrate the position of the most enriched genes on this ranked genes GSEA analysis). d2, d5: genes enriched or depleted in 3-genes positive versus negative OSCC samples from dataset GSE41613 with absolute fold change > 2 and *P* value < 0.01 (respectively, *n* = 48 genes or *n* = 112 genes). d3, d6: genes enriched or depleted in 3-genes positive versus negative OSCC samples from dataset TCGA-HNSC with absolute fold change > 2 and *P* value < 0.01) (respectively, *n* = 43 genes or *n* = 49 genes). **e** GSEA plots showing the enrichment/depletion of genesets indicative of sensitivity or resistance to therapeutics of aggressive 3-genes positive versus negative OSCC tumours from the GEO GSE41613 (1st column) and TCGA-HNSC (2nd column) datasets, FaDu cell line expressing high versus low levels of CCNA1 after experimental downregulation of *CCNA1* (3rd column), and HPV-negative versus positive tumours from the TCGA-HNSC dataset (4th column). The following selection of three genesets are shown whose enrichment suggest (i) resistance to doxorubicin (5-fluorouracil): genes upregulated in doxorubicin-resistant vs -sensitive gastric cancer cell lines [28]), (ii) a potential sensitivity to Aplidin, a marine-derived compound with potential anticancer properties: genes downregulated in the MM1S cells (multiple myeloma) after treatment with aplidin [29], (iii) a sensitivity to Dasatinib, a multitargeted kinase inhibitor: genes whose expression positively correlated with sensitivity of breast cancer cell lines to Dasatinib: [30]). The details about the genesets are available on the MsigDB website (http://www.gsea-msigdb.org/gsea/msigdb/search.jsp). For detailed enrichment score values and composition of the genesets, please refer to Supplementary Table S6. Supplementary Fig. S9 shows more GSEA plots characterising the molecular profile of aggressive forms of OSCCs.

For this purpose, we identified an OSCC cell line, FaDu (ATCC), which expressed significant levels of *CCNA1*, and analysed the effects of a moderate knockdown of *CCNA1* on the proliferation abilities of the cells by FACS analysis of the cell cycle, as well as on the transcriptomic signature by RNAseq analysis. The partial knockdown of CCNA1 was evidenced by western blots (Fig. 5a), and the experimental results demonstrate that CCNA1 is clearly involved in the proliferative ability of the OSCC cells since its downregulation had an inhibitory effect on the cell cycle (Fig. 5b).

Interestingly, the comparison between the whole genome expression profile of 3-genes positive versus negative tumours and that of the FaDu OSCC cell line over-expressing or not *CCNA1* shows that these two signatures are highly similar (Fig. 5c, d, GSEA plots shown in Supplementary Fig. S9C). This observation suggests that by downregulating CCNA1 we obtain a shift of the transcriptomic signature associated with aggressive OSCC tumours towards that of the less aggressive forms of OSCC, and therefore that CCNA1 could be a good target for therapeutic approaches in 3-genes positive tumours.

In addition, the expression profile of aggressive 3-genes positive OSCC tumours, as well as that of OSCC cells expressing high levels of CCNA1 (FaDu cells WT as compared to *CCNA1* KD cells), also show a significant upregulation of genes signatures associated with resistance to doxorubicin (5-fluorouracil) (genes upregulated in doxorubicin-resistant versus -sensitive gastric cancer cell lines [28]), but a potential sensitivity to Aplidin, a marine-derived compound with potential anticancer properties [29]), and most interestingly a sensitivity to Dasatinib, a multitargeted kinase inhibitor [30] (Fig. 5e).

Finally, we compared the signature of HPV-negative (poor prognosis) versus -positive (longer survival) tumours (Fig. 4d, e). Some of these features were shared by both signatures, including the enrichment in HRAS, TNFA/NFKB and EGF signatures (Supplementary Fig. S9D), as well as the signature associated with the Dasatinib sensitivity (Fig. 5e, 4th column). However, the genesets related to proliferation and cell cycle opposed the 3-genes positive and the HPV-negative aggressive cells, with the former being enriched and the latter depleted in these genesets (Supplementary Fig. S9, compare ABC panels with D panels). This observation suggests that the high proliferative nature of aggressive 3-genes positive OSCC is not shared by the HPV-negative OSCC, where aggressiveness is associated with a probably high contingent of dormant cells.

## Discussion

Much effort has been put by the medical and scientific communities into the discovery of informative biomarkers easily detectable by pathologists as part of diagnosis protocols.

The approach described in ref. [6], based on REMARK guidelines and good practice in statistics, offers clear guidance. Indeed, the authors aim at increasing the efficiency of marker discovery by using strict criteria for the selection of studies as well as for ranking the level of evidence of the identified biomarkers, ranging between exploratory, to validated and finally clinically relevant markers. These guidelines also inspired our work and incited us to apply highly selective criteria to our marker discovery approach to increase the robustness of our markers and to validate them as informative IHC-detectable markers.

Within this frame, our approach is novel for several reasons. First, since it focuses on genes/proteins which are normally silent or expressed at very low levels in non-germline adult tissues, it is possible to define a threshold of signal above which they are considered expressed, at the mRNA level or at the protein level. This situation considerably increases our chances to obtain consistent results regardless of the technology or whether the expression is measured from mRNA or proteins species. Second, by ensuring that the survival probability was not only correlated to the expression level but also that a range of thresholds would efficiently separate patients with different prognoses when using mRNA-based data, we also increased our chances to succeed in obtaining consistent results with immunohistochemistry detection of the corresponding proteins. A third important characteristic of our approach is to propose combinations of a small number of genes/proteins as the basis of our test. Indeed, a combination of several well-chosen biomarkers enables to increase the robustness of the test.

The main limitation of our work is the relatively small size of our own independent validation cohort, 66 samples analysed retrospectively, which was the cohort where the ectopic activations were measured by immunohistochemistry. However, although the numbers of patients are limited, the association between the detection of the three proteins and prognostic is clearly significant (Fig. 3c). The fact that the same association with prognostic was observed in this cohort as in the two other unrelated cohorts where abnormal activations were detected using mRNA measurements, is highly supportive of a strong and consistent association between the abnormal expression of these three proteins and OSCC aggressiveness.

None of the 15 genes that we identified here to be associated with OSCC prognostic was found in the lists of genes from the two meta-analyses of oral tongue squamous cell carcinoma marker candidates [5, 6]. However, a systematic screen of the literature, shows that the expression of 5 out of the 15 genes, including AREG, CCNA1, CDKN3, MMP10 and SUV39H2, have already been reported to be associated with survival probabilities in OSCC or HNSC (see Supplementary Table S7; for AREG see refs. [24, 31–34]; for CCNA1 see refs. [35, 36]; for CDKN3 see refs. [37, 38]; for MMP10 see refs. [39–41]; for SUV39H2 see ref. [42]). The expression levels of an additional three genes, NUP155, TDO2 and TRIP13, have been found correlated with shorter survival in oesophageal squamous carcinomas, as well as with other cancers ([43–45] for NUP155, TDO2 and TRIP13, respectively). For DDX20/GEMIN3, no association between expression and OSCC had yet been reported, but a specific single-nucleotide variant has been found associated with the recurrence of oropharyngeal cancer patients after definitive radiotherapy [46].

The three genes themselves are normally expressed in the placenta (*AREG*) or in male germ cells (*CCNA1* and *DDX20*) (Supplementary Fig. S10) but silent in non-germline adult tissues. Interestingly, the corresponding proteins CCNA1, AREG or DDX20 have been individually found involved in a large range of solid tumours, including head and neck carcinoma.

In various cancer types, including Head and neck carcinomas, Amphiregulin (AREG) overexpression has been involved in therapeutic failure and resistance to anti-EGFR therapies [32, 47] or to vincristine, a microtubule-destabilising agent [34].

DEAD (Asp–Glu–Ala–Asp) box polypeptide 20 (DDX20, also known as GEMIN3 or DP103), a member of the DEAD-box protein family, encodes an ATPase-dependent RNA helicase. A deregulated DDX20 activity has been reported correlated with aggressive behaviour and metastatic potential in several tumours, including prostate cancer [22], hepatocellular carcinoma [48] and breast cancers [49].

CCNA1 is a cyclin-dependent protein serine/threonine kinase mainly expressed in testis. It belongs to the highly conserved cyclin family, whose members are characterised by a periodicity in abundance through the cell cycle. CCNA1 is involved in cell cycle progression and its silencing in leukaemia cells inhibits cell growth [50].

The expression of CCNA1 has been found associated with poor prognosis in various cancers, including bladder urothelial carcinomas [23], oesophageal squamous cell carcinoma [36], as well as in head and neck cancer [35, 51].

Its largely predominant expression in the testis, the existence of targetable kinase activity and its recurrent association with aggressive tumours suggests that it could be used as a specific therapeutic target. The experimental partial knockdown of the corresponding gene in the FaDu OSCC cell line supports this hypothesis since it resulted not only in a cell cycle inhibition (shown in Fig. 5b), but also in the downregulation of the whole genome expression profile associated with the aggressive 3-genes positive tumours (Fig. 5c, d, Supplementary Fig. S9C and Fig. 5e, 3rd column). It is also of note that CCNA1 overexpressing OSCC cells are highly enriched in the signature of Dasatinib sensitivity, which suggests that the CCNA1 kinase could be one of the major targets of Dasatinib in CCNA1 high expressing cells.

Altogether this study not only led to the discovery of a set of three proteins which in combination provide an easy-to-use immunohistochemistry set of biomarkers for the early detection of the aggressive forms of OSCC but also revealed a new level of tumour heterogeneity among the HPV-positive OSCC. Integrative analysis of the molecular signature characterising this subset of OSCC strongly suggests that specific molecular pathways are involved. Furthermore, the downregulation of one of the three proteins, CCNA1, induces a decrease in proliferating cells as well as a downregulation of the whole-genome expression profile from aggressive to less aggressive OSCC cells, suggesting that this kinase could be used as a target for the future development of new therapeutic strategies in cases that do not respond or escape existing treatments.

Prospective studies in large cohorts of patients will be required to confirm this triple IHC test as a reliable universal marker of OSCC aggressiveness. Functional studies in appropriate cells and mouse models will enable researchers to define new therapeutic protocols, which could improve survival of the 3-genes-positive OSCC patients, upgrading this 3-genes/proteins IHC detection to the status of a “companion” test, predicting the patients’ response to specifically dedicated treatments.

## Supplementary information

Supplemental methods figures and legends

Supplemental tables

REMARK checklist

authors list agreement

Reproducibility checklist

## Data Availability

The RNAseq data obtained from FaDu wild-type or after knockdown of *CCNA1* have been made publicly available on the GEO repository: https://www.ncbi.nlm.nih.gov/geo/query/acc.cgi?acc=GSE171506. All other original data are made available in the main manuscript and supplemental data.
