# Supplementary material for: The combined detection of Amphiregulin, Cyclin A1 and DDX20/Gemin3 expression predicts aggressive forms of oral squamous cell carcinoma
Source: Br J Cancer. 2021 Jul 21;125(8):1122–34. doi: 10.1038/s41416-021-01491-x (PMC8505643; doi:10.1038/s41416-021-01491-x)
Supplement: Supplementary file 1 — Supplemental methods figures and legends [file 41416_2021_1491_MOESM1_ESM.pdf]

The combined detection of Amphiregulin, Cyclin A1 and DDX20/Gemin3 expression predicts aggressive forms of Oral Squamous Cell Carcinoma

Ekaterina Bourova-Flin[1], Samira Derakhshan[2], Afsaneh Goudarzi[3], Anne-Laure Vitte[1], Florent Chuffart [1], Saadi Khochbin[1], Sophie Rousseaux[1]*, Pouyan Aminishakib[2]*

*Corresponding author Email: aminishakib@tums.ac.ir (PA); sophie.rousseaux@univ-grenoble-alpes.fr (S.R.)

Affiliations:

[1] CNRS UMR 5309/INSERM U1209/University Grenoble-Alpes/Institute for Advanced Biosciences, La Tronche, France.
[2] Oral and Maxillofacial Pathology Department, School of Dentistry, Tehran University of Medical Sciences, Tehran, Iran.
[3] Department of Clinical Biochemistry, School of Medicine, Shahid Beheshti University of Medical Sciences, Tehran, Iran.

The authors declare no potential conflicts of interest.

Running title
Three-proteins based test predicts survival in OSCC

Supplemental Methods

Data

Cohort 1. Training cohort – Microarrays.

In this study, we used the public dataset GSE41613 (Affymetrix Human Genome U133 Plus 2.0 Array) as the training cohort for biomarker discovery. This cohort contains 97 samples annotated with demographic (age group, gender), clinical (tumour stage group, HPV status) and overall survival data. The follow-up ranges from 52.6 to 85.0 months with median survival time at 65.0 months. The number of events (deaths) is 51, corresponding to 52.6% of the patients.

Cohort 2. Validation cohort – RNAseq.

The second public cohort TCGA-HNSC of RNAseq data was used as a validation dataset. This cohort contains 467 samples with available overall survival information. Several demographic and clinical data are also provided, which include age, gender, TNM status, TNM stage, grade, HPV status and anatomic site. The follow-up ranges from 0.4 to 182.7 months with median survival time at 57.3 months. The number of events (deaths) is 197, corresponding to 42.2% of the patients.

Cohort 3. Validation cohort – Immunohistochemistry.

For validation by immunohistochemistry, we used our retrospective cohort of 66 OSCC patients with detailed demographic and clinical annotations (Supplementary Table S4). The follow-up ranges from 3 to 262 months with median survival time at 193 months. The number of events (deaths) is 14, corresponding to 21.2% of the patients.

Sample size

To design our validation cohort of OSCC patients for immunodetection (cohort 3), we estimated its size by using the formula for proportional hazards model of Latouche and collaborators (Latouche *et al*, 2004). We used Python package "lifelines.statistics" and the function "sample_size_necessary_under_cph" within the package for sample size calculation. The probabilities of events in the groups were estimated from the results obtained in the training cohort GSE41613 (cohort 1): 36.4% of events in the "0-1" group and 74.2% in the "2-3" group. An overall sample size of 66 subjects in cohort 3 achieves 80.0% power and 0.05 significance

level for hazard ratios greater than 2.7. The actual hazard ratio measured in our validation cohort was 4.15 (see Fig.3C).

Biomarker discovery

An overview of the biomarker discovery strategy is presented in Fig.1A. The steps are detailed below.

1- Mining publicly available transcriptomic data for the discovery of OSCC prognostic candidate markers

The overall strategy is an adapted and updated version of the data mining approach thoroughly described in (Rousseaux *et al*, 2013a). The purpose of this dedicated approach is to mine publicly available transcriptomic data to detect the abnormal expression of genes that should not be normally expressed and find correlations between these activations and survival. This approach was updated to exploit RNAseq data.

It is based on the metanalysis of expression data of normal tissues and tumours i/ first to identify genes whose expression is specific or highly predominant in one tissue type, most frequently male germ cells, embryonic stem cells or placenta, and silent (or with very low levels of expression) in normal non-germline tissues ii/ second to detect their activation in individual OSCC samples and iii/ third to correlate these abnormal expressions with clinical data, particularly with survival data.

Establishment of a list of tissue-predominant genes.

RNAseq data from various normal human tissues of publicly available GTEx dataset (https://gtexportal.org) and E-MTAB-1733 dataset (https://www.ebi.ac.uk/arrayexpress/) were used to identify genes with an expression pattern largely predominant in one tissue type or group. For this purpose, we first log-transformed available RPKM data by applying log2(1+RPKM) and then measured a mean expression level corresponding to each tissue for each gene (i.e. average log-transformed RPKM by tissue). To identify tissue-predominant genes, we systematically looked if a gene had a more important average expression in one particular tissue compared to all other tissues, by using a z-score method to detect outliers with a threshold of 3.7 for 40 tissue groups.

This investigation identified more than 3000 genes predominantly expressed in testis or placenta, and not expressed or expressed only at low levels in other adult somatic tissues.

Among these tissue-predominant genes, 1088 were available in GSE41613 dataset and 2084 in TCGA-HNSC dataset.

Detection of abnormal gene expression in OSCC and correlation with prognosis.

The expression of testis and placenta-predominant genes in OSCC was detected using a transcriptomic dataset of OSCC tumours obtained with the Affymetrix Human Genome U133 Plus 2.0 Array technology (GSE41613, n=97, patients with survival data). The raw data (.CEL files) from this dataset was normalized together with data from normal head and neck tissues obtained in the same technology (GSE3526, GSE6791 and GSE7307, total number of controls is n=61).

Our goal was to identify genes for which an abnormal expression would negatively affect patients' prognosis, with the aim to develop a test based on the detection of the expression of the encoded proteins by immunohistochemistry (IHC). Our strategy included three steps.

1. First, for each tissue-predominant gene, we tested the association between expression and survival probability in a univariate Cox proportional hazard model and selected the genes for which expression level was significantly associated with overall survival (Cox model p-value < 0.05).
2. In the second step, for each gene selected in step 1 we checked if it was possible to define thresholds that could stratify patients into two groups with significantly different prognosis. This step was performed in order to increase our chances to be able to also find a correlation between survival and the activation of the encoded protein detected by immunohistochemistry, a semi-quantitative approach to measure protein expression. For this purpose, we tested all possible thresholds in the range from the average value in normal samples up to the 80th percentile of expression in tumour samples, with a step of one percentile. When the signal in normal samples was too low, we used the 20th percentile expression in tumour samples as the minimal threshold. All thresholds were analysed using logrank statistical test between two groups of low and high expression. A threshold was considered as significant if the corresponding logrank p-value < 0.05. When several significant thresholds were present, we selected one reference threshold corresponding to the minimal logrank p-value.
3. Finally, we calculated the range of significant thresholds obtained for each gene. We selected only the genes for which the interval of significant thresholds was greater than 50%. This constraint ensured a robust threshold: a small modification of the threshold would not significantly change the result.

2- Stratification of patients using a subset of three genes, design and first validation of a three-genes based prognostic stratifying system using an independent cohort with transcriptomic data

Following the method described above, we identified 15 candidate genes that were significantly associated with overall survival and had a stable threshold. We kept three of them, *AREG, CCNA1* and *DDX20*, based on the availability of antibodies for the immunohistochemistry technique. We then used these three genes in combination as a classifier.

In order to test the ability of three genes, *AREG, CCNA1* and *DDX20*, in combination to predict prognosis, the patients of the training cohort (GSE41613 Affymetrix n=97) were grouped according to the sum of positive expressions for these three genes and survival probabilities were compared between the groups of patients.
This 3-genes based classifying system was further validated in an independent dataset of OSCC patients, RNAseq data from the TCGA-HNSC study corresponding to 467 patients with overall survival data. The threshold was calculated following the same procedure as for GSE41613 Affymetrix data.

3- Stratification of the OSCC patients according to the protein/antigenic signature using IHC on tumour sections in our cohort of OSCC patients
The 4th step of our approach was a 2nd validation of our prognostic test in our cohort of OSCC patients. It is entirely described in the main text.

***4- CCNA1* knock-down in the OSCC cell line FaDu (ATCC), FACS analysis, RNAseq data generation and differential expression analysis.**

FaDu cell line culture and CCNA1 Knock down
FaDu cell line (ATCC- HTB-43) were subcultured in EMEM complete medium. *CCNA1* Knock Down was performed using the Lipofectamin RNAiMax transfection reagent (INVITROGEN) according to the manufacturer's protocol. The siRNA sequence targeting CCNA1 was GAACCUGGCUAAGUACGUA, and the RNA sequence used as Control was the siRNA pGL3 luciferase control (Eurogentec reference SR-CL011-005).

FACS analysis of cell-cycle

Cells were trypsinised 48 hours after transfection. One million cells were washed in cold PBS, resuspended in cold 70 % EtOH Overnight at 4°C, washed once again in cold PBS, suspended in 500 µl of PBS with 0.01% Triton, 0.05 mg/ml Propidium Iodide, 0.2 mg/ml RNAse A, incubated for 30 minutes at 37°C, and then immediately subjected to FACS analysis with the Accuri C6 Flow Cytometer (BD).

RNA-seq generation and differential expression analysis

RNA extractions were performed from control and *CCNA1* knock down FaDu cells in independent triplicates for each condition. RNA was extracted from each sample directly after cell harvesting with TRIZOL, following the manufacturer's protocol (AMBION). RNA pellets were dissolved in RNAse-free water, and then cleaned up on QIAGEN columns using the RNA Clean Up protocol of RNeasy Mini Kit (QIAGEN), which includes an on column-DNase treatment of 15 minutes. RNA was eluted with 40 µl of RNAse-free water and its concentration measured with Nanodrop.

RNAseq was subcontracted to BGI Tech Solution (Hong Kong). For each sample, 1 µg RNA was used for libraries preparation with LncRNA library(H/M/R) according to the manufacturer's instructions and sequenced on a DNBSEQ PE100 Eukaryotic Long Non-Coding RNA platform.

The sequenced reads were aligned from raw sequence fastq data using STAR v2.5.2b software on UCSC hg38 reference genome. The aligned reads were normalized using the R bioconductor package DEseq2 (http://bioconductor.org/packages/3.12/bioc/html/DESeq2.html) and log transformed using the R bioconductor package DEseq2 (http://bioconductor.org/packages/3.12/bioc/html/DESeq2.html).

Differential transcriptomic analyses were performed to identify genes significantly up-and down regulated between two conditions using thresholds of Student t-test p-value <0.01 and fold change absolute value of 2.

The raw data and normalized read counts have been deposited on the GEO website (https://www.ncbi.nlm.nih.gov/geo/query/acc.cgi?acc=GSE171506).

5- Molecular characterization of aggressive forms of OSCC: differential analysis of transcriptomes between aggressive tumours and the others, followed by a GSEA approach.

The possibility to efficiently stratify OSCC tumours according to their level of aggressiveness and the availability of genome-wide transcriptomic data, was exploited here to explore the underlying molecular profiles associated with high risk of treatment resistance and/or post-treatment recurrence. A differential transcriptomic analysis was performed to identify genes differentially expressed between OSCC samples positive and negative for the 3 genes. A signature corresponding to the ratios of expression values in positive versus negative OSCC samples was then used to carry out a Gene set enrichment analysis (GSEA) using the GSEA software (available https://www.gsea-msigdb.org/gsea/downloads.jsp, Subramanian et al. 2005, PNAS 102, 15545-15550, PMID: 16199517; Mootha, et al. 2003, Nat Genet 34, 267-273, PMID: 12808457) on the collections of gene sets made available by the Broad Institute (GSEA tool: MSigB: http://software.broadinstitute.org/gsea/msigdb/index.jsp).

Using a similar GSEA approach the signatures of genes differentially expressed between HPV negative versus positive samples, as well as of genes up-and down-regulated in the FaDu OSCC cell line after Knock Down of *CCNA1* compared to FaDu cells treated by siRNA pGL3 luciferase control were also studied and compared with the whole genomic expression signature 3-genes positive versus negative OSCC patients’ samples.

Supplementary figures

Supp. Fig. S1. Expression levels of each the 3 genes in normal and tumour samples from the publicly available OSCC cohort GSE41613, Kaplan-Meier curves showing their association with survival probability, and definition of intervals of significant thresholds associated with prognosis.

For each gene we considered a range of possible thresholds (from 20th to 80th percentile) and successively tested each threshold for its ability to discriminate between two groups of tumours (of low and high expression values) corresponding to significantly different survival probabilities.

The plots of the left show the distribution of expression values in normal and tumour samples. For each gene, the selected threshold is shown as a dashed green line whereas the dashed grey line corresponds to the mean value of signal in non-tumour samples. The grey solid area corresponds to the interval of all tested thresholds.

The middle plots show Kaplan-Meier survival curves for patients with low (in blue) and high (in red) expressions of the corresponding gene, respectively below or above the selected threshold.

The plots on the right show the distribution of p-values from univariate Cox statistical tests (y-axis) obtained for all tested thresholds (x-axis). The significant p-values (p-value<0.05) are plotted in green, whereas nonsignificant p-values are represented in grey. The selected threshold is marked by a star. For each gene, the interval of significant thresholds was defined as the proportion of significant thresholds over all tested thresholds.

Supp. Fig. S1

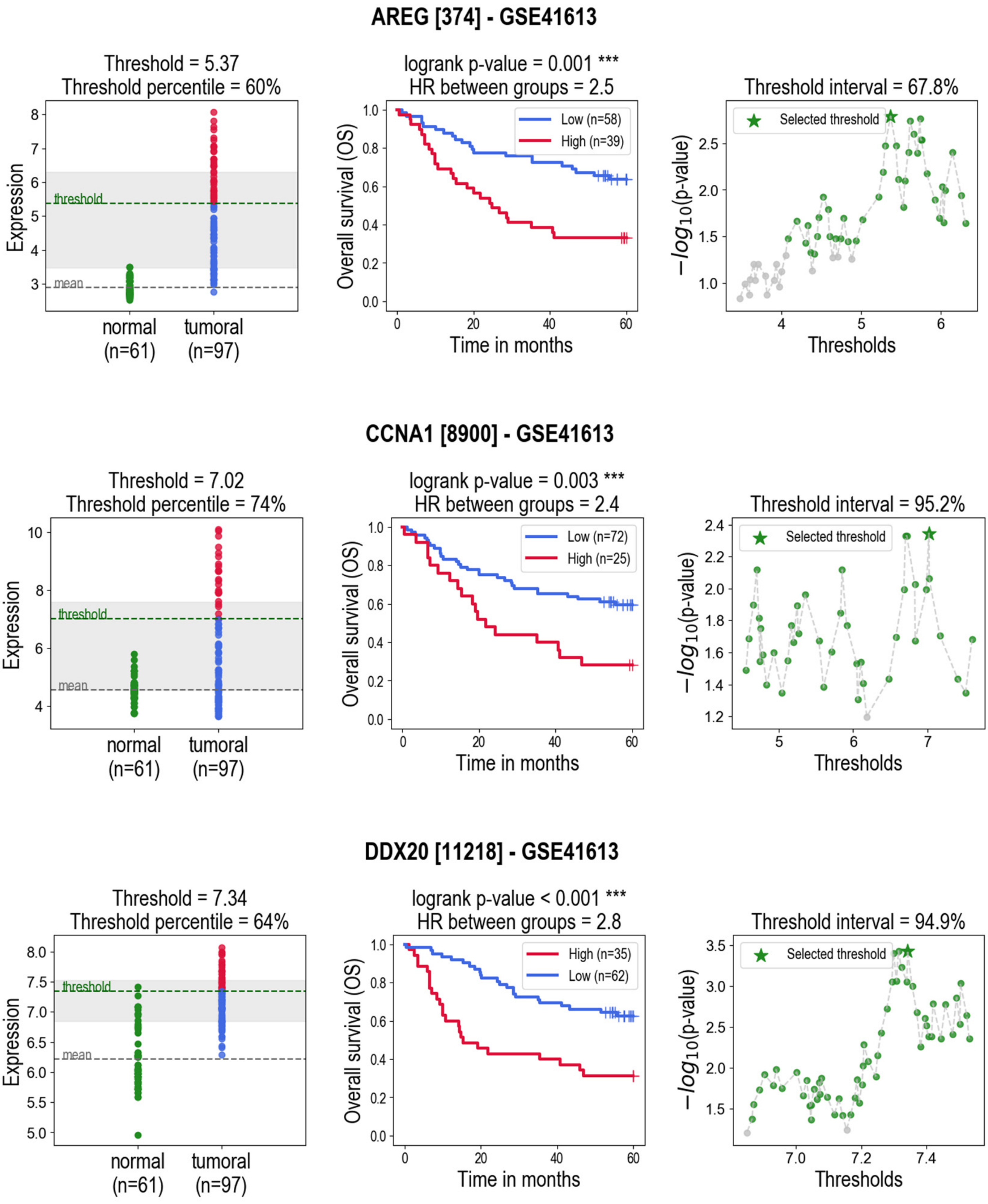
AREG [374] - GSE41613
Threshold = 5.37
Threshold percentile = 60%
Expression
threshold
mean
normal (n=61)
tumoral (n=97)
logrank p-value = 0.001 ***
HR between groups = 2.5
Overall survival (OS)
Low (n=58)
High (n=39)
Time in months
Threshold interval = 67.8%
Selected threshold
−log10(p-value)
Thresholds
CCNA1 [8900] - GSE41613
Threshold = 7.02
Threshold percentile = 74%
Expression
threshold
mean
normal (n=61)
tumoral (n=97)
logrank p-value = 0.003 ***
HR between groups = 2.4
Overall survival (OS)
Low (n=72)
High (n=25)
Time in months
Threshold interval = 95.2%
Selected threshold
−log10(p-value)
Thresholds
DDX20 [11218] - GSE41613
Threshold = 7.34
Threshold percentile = 64%
Expression
threshold
mean
normal (n=61)
tumoral (n=97)
logrank p-value < 0.001 ***
HR between groups = 2.8
Overall survival (OS)
High (n=35)
Low (n=62)
Time in months
Threshold interval = 94.9%
Selected threshold
−log10(p-value)
Thresholds

Supp. Fig. S2. Kaplan Meier survival curves showing the association between the expression of each of the selected 15 genes and the OSCC patients from the training cohort GSE41613.

The genes were selected according to the criteria detailed in Fig 1A and its legend (Cox p-value < 0.05, Logrank p-value (between groups) <0.05, and interval of significant thresholds >50%). The hazard ratio between groups is shown here but it was not used for selection (also see Supp. Table S2A).

Supp. Fig. S2

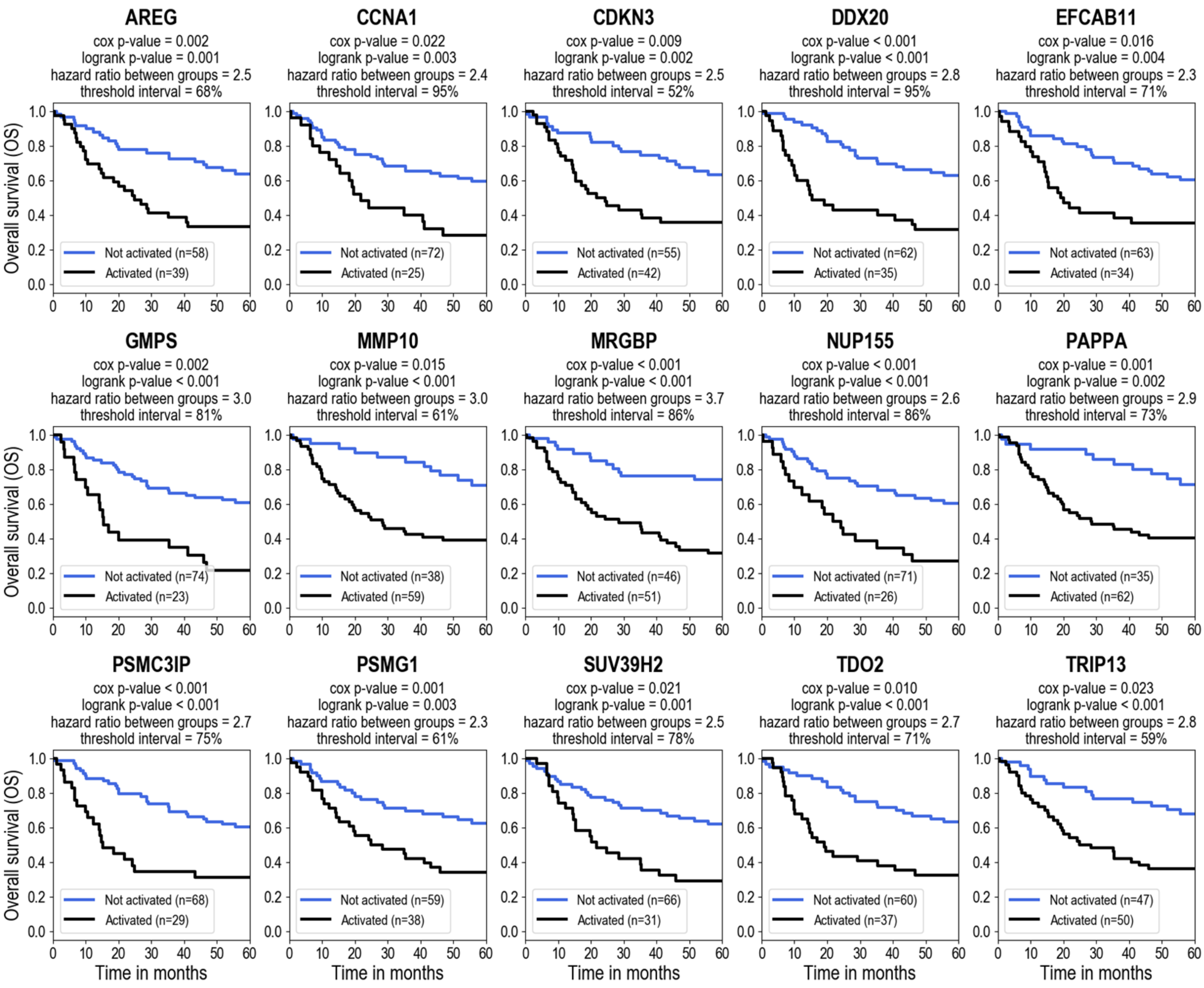

AREG
cox p-value = 0.002
logrank p-value = 0.001
hazard ratio between groups = 2.5
threshold interval = 68%
Not activated (n=58)
Activated (n=39)
CCNA1
cox p-value = 0.022
logrank p-value = 0.003
hazard ratio between groups = 2.4
threshold interval = 95%
Not activated (n=72)
Activated (n=25)
CDKN3
cox p-value = 0.009
logrank p-value = 0.002
hazard ratio between groups = 2.5
threshold interval = 52%
Not activated (n=55)
Activated (n=42)
DDX20
cox p-value < 0.001
logrank p-value < 0.001
hazard ratio between groups = 2.8
threshold interval = 95%
Not activated (n=62)
Activated (n=35)
EFCAB11
cox p-value = 0.016
logrank p-value = 0.004
hazard ratio between groups = 2.3
threshold interval = 71%
Not activated (n=63)
Activated (n=34)
GMPS
cox p-value = 0.002
logrank p-value < 0.001
hazard ratio between groups = 3.0
threshold interval = 81%
Not activated (n=74)
Activated (n=23)
MMP10
cox p-value = 0.015
logrank p-value < 0.001
hazard ratio between groups = 3.0
threshold interval = 61%
Not activated (n=38)
Activated (n=59)
MRGBP
cox p-value < 0.001
logrank p-value < 0.001
hazard ratio between groups = 3.7
threshold interval = 86%
Not activated (n=46)
Activated (n=51)
NUP155
cox p-value < 0.001
logrank p-value < 0.001
hazard ratio between groups = 2.6
threshold interval = 86%
Not activated (n=71)
Activated (n=26)
PAPPA
cox p-value = 0.001
logrank p-value = 0.002
hazard ratio between groups = 2.9
threshold interval = 73%
Not activated (n=35)
Activated (n=62)
PSMC3IP
cox p-value < 0.001
logrank p-value < 0.001
hazard ratio between groups = 2.7
threshold interval = 75%
Not activated (n=68)
Activated (n=29)
PSMG1
cox p-value = 0.001
logrank p-value = 0.003
hazard ratio between groups = 2.3
threshold interval = 61%
Not activated (n=59)
Activated (n=38)
SUV39H2
cox p-value = 0.021
logrank p-value = 0.001
hazard ratio between groups = 2.5
threshold interval = 78%
Not activated (n=66)
Activated (n=31)
TDO2
cox p-value = 0.010
logrank p-value < 0.001
hazard ratio between groups = 2.7
threshold interval = 71%
Not activated (n=60)
Activated (n=37)
TRIP13
cox p-value = 0.023
logrank p-value < 0.001
hazard ratio between groups = 2.8
threshold interval = 59%
Not activated (n=47)
Activated (n=50)
Overall survival (OS)
Time in months
0.0 0.2 0.4 0.6 0.8 1.0
0 10 20 30 40 50 60

Supp. Fig. S3. Kaplan Meier survival curves illustrating the correlation between clinical parameters gender (upper panel) and stage (lower panel) and prognosis of OSCC patients from the GSE41613 cohort (n=97).
This cohort was used as our training dataset for the selection of prognostic associated ectopic gene activations.

Supp. Fig. S3

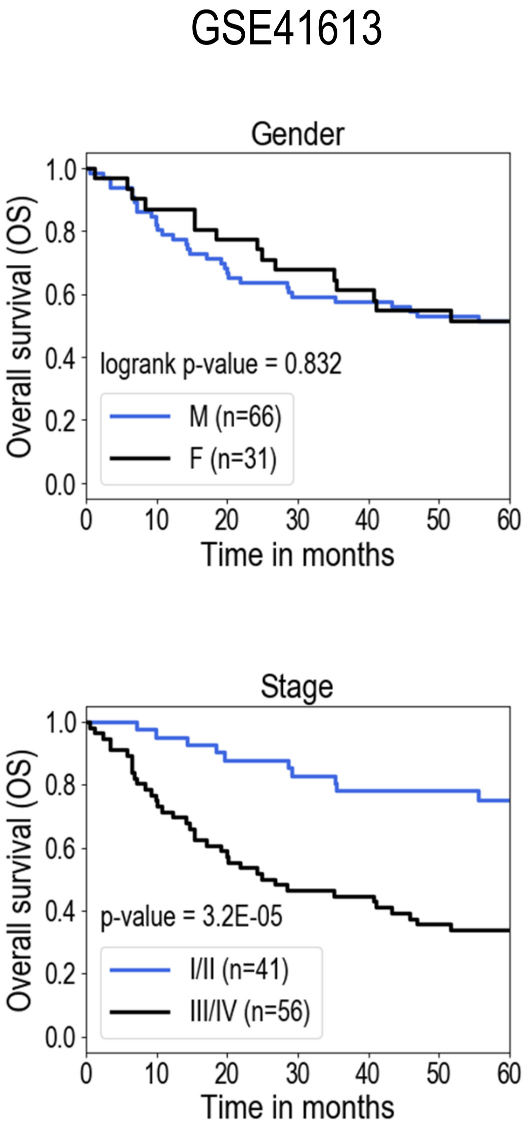
GSE41613
Gender
Overall survival (OS)
1.0
0.8
0.6
0.4
0.2
0.0
logrank p-value = 0.832
M (n=66)
F (n=31)
0
10
20
30
40
50
60
Time in months
Stage
Overall survival (OS)
1.0
0.8
0.6
0.4
0.2
0.0
p-value = 3.2E-05
I/II (n=41)
III/IV (n=56)
0
10
20
30
40
50
60
Time in months

Supp. Fig. S4. Kaplan Meier survival curves showing the association between the expression of each of the 3 genes which were finally selected and the HNSC patients from the validation cohort TCGA-HNSC.
When the genes are considered individually, the association between expression and prognostic reaches significance for only two of them, *AREG* and *CCNA1* whereas for *DDX20*, although the patients with a tumour expressing the gene tend to have shorter survival than those with tumour not expressing the gene, this association does not reach significance in this second cohort. The detailed analysis of correlations between expression of the 15 preselected genes and survival probabilities in the TCGA-HNSC cohort is shown in Supp. Table S2B.

Supp. Fig. S4

3 selected genes in TCGA-HNSC

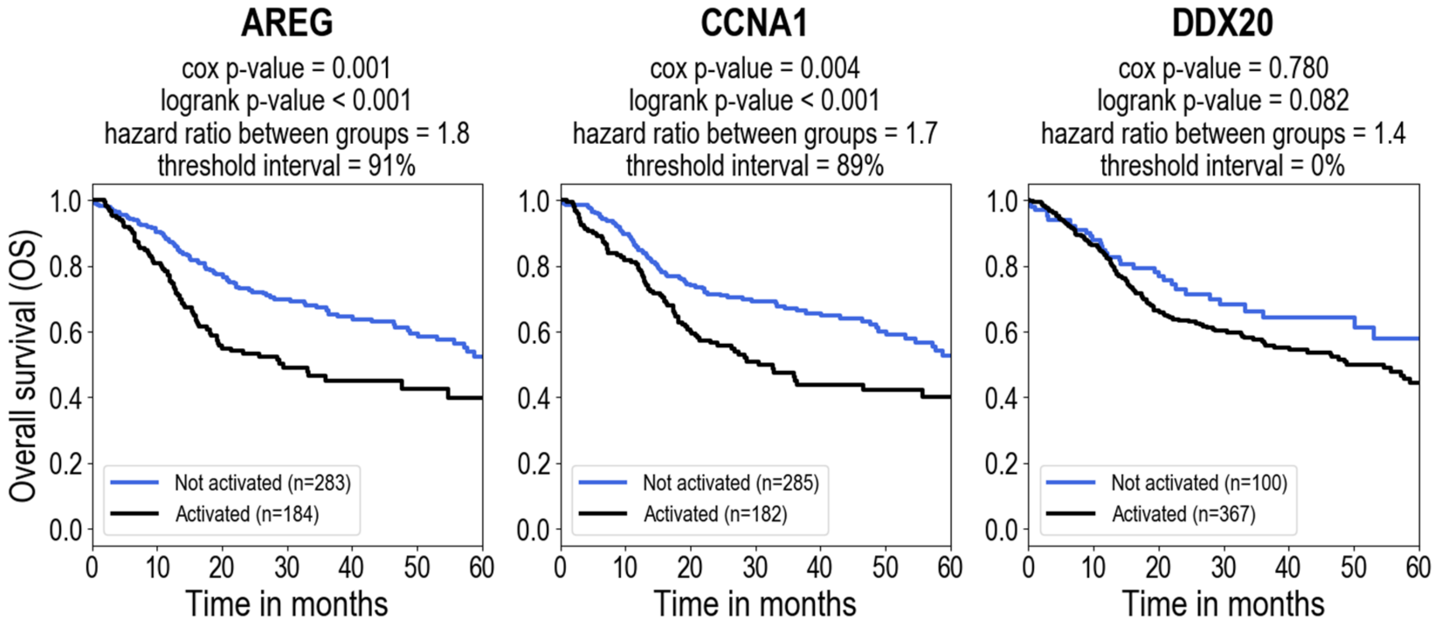

Supp. Fig. S5. Association of clinical and biological parameters with prognosis of HNSC patients from the TCGA HNSC cohort (n=467)

Kaplan Meier survival curves illustrating HNSC patients' survival according to

A. Gender: male (M) (black) versus female (F) (blue)

B. Grade: grade I (blue) versus grades II and III (black)

C. TNM: patients with tumour size staged T1 or T2 and lymph nodes invasion staged N0 or N1 (early stage, blue) compared with patients with tumour size >= T3 or N stage >= N2 (late stage, black)

D. Tumour stage: I, II, III and IVA and IVB (as indicated)

E. HPV status (p16): positive (blue) versus negative (black)

F. Anatomic site: Tonsil or Base of tongue TBOT (blue) versus other sites (black)

Supp. Fig. S5

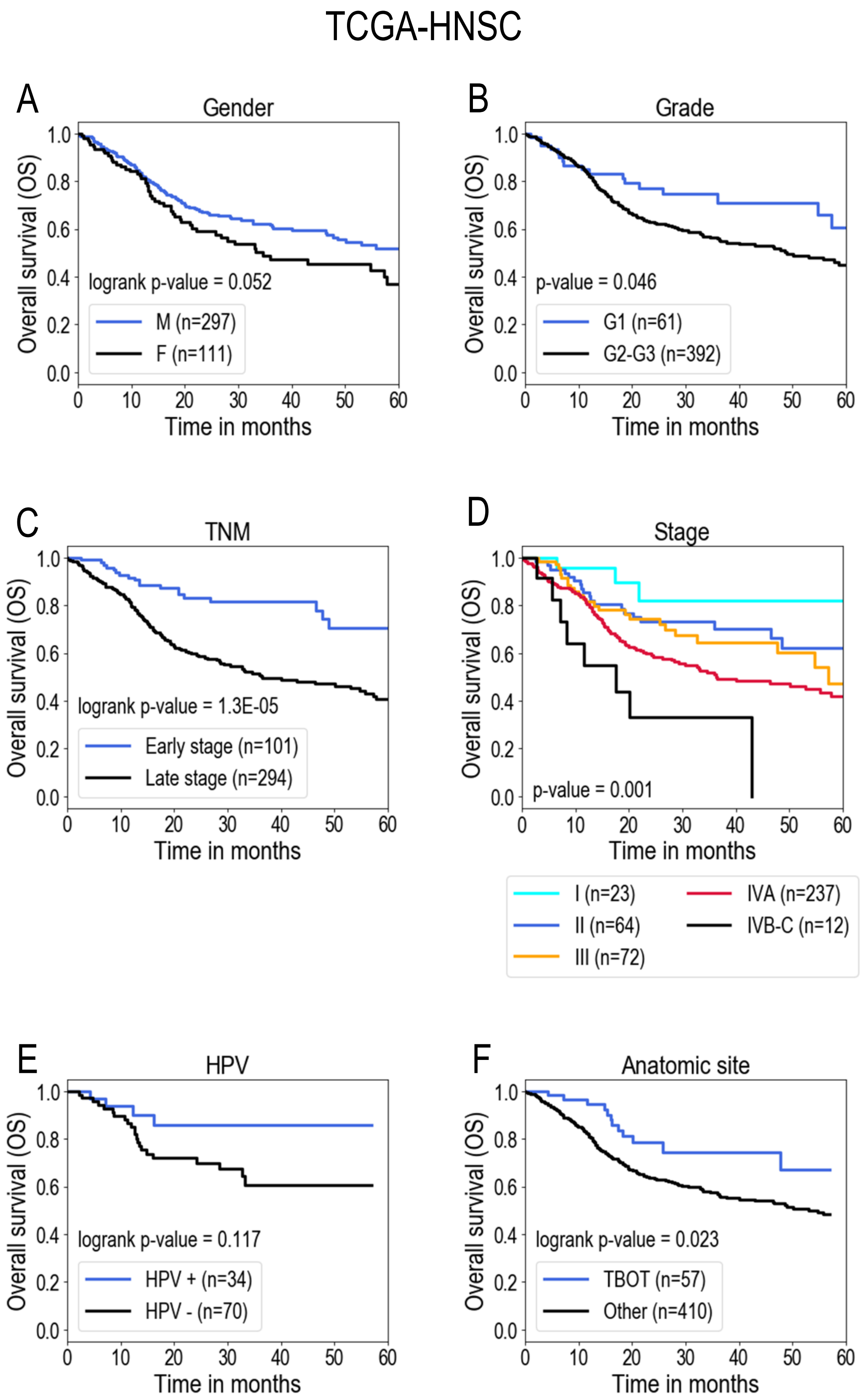
TCGA-HNSC
A
Gender
Overall survival (OS)
1.0
0.8
0.6
0.4
0.2
0.0
logrank p-value = 0.052
M (n=297)
F (n=111)
0
10
20
30
40
50
60
Time in months
B
Grade
p-value = 0.046
G1 (n=61)
G2-G3 (n=392)
C
TNM
logrank p-value = 1.3E-05
Early stage (n=101)
Late stage (n=294)
D
Stage
p-value = 0.001
I (n=23)
II (n=64)
III (n=72)
IVA (n=237)
IVB-C (n=12)
E
HPV
logrank p-value = 0.117
HPV + (n=34)
HPV - (n=70)
F
Anatomic site
logrank p-value = 0.023
TBOT (n=57)
Other (n=410)

Supp. Fig. S6 – Kaplan Meier survival curves showing the association of clinical and biological parameters with prognosis of the 66 OSCC patients from our cohort: overall survival over five years (60 months) according to (A) the year of diagnosis, (B) gender, (C) grade and (D) tumour T stage.

Supp. Fig. S6

our OSCC cohort

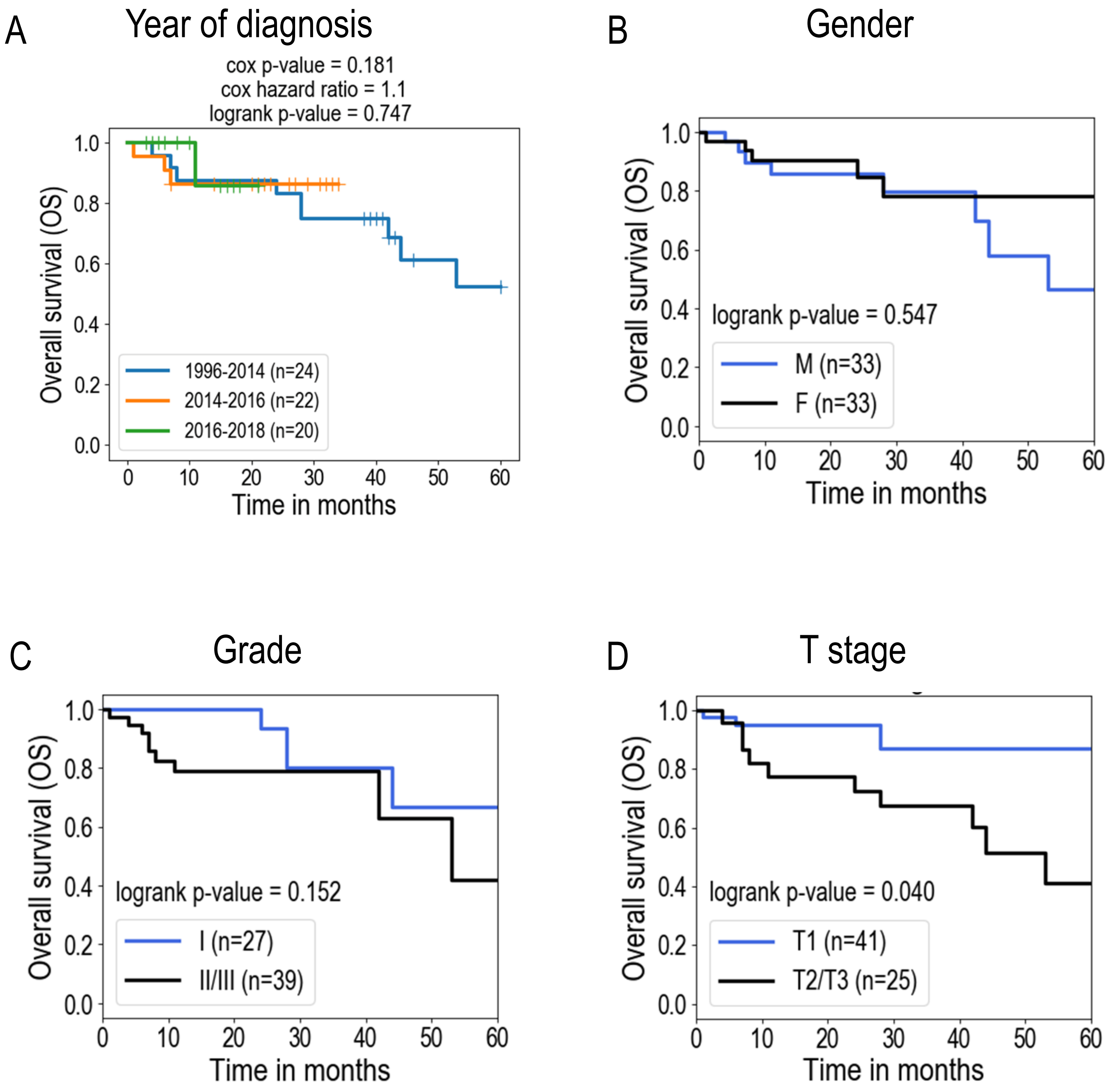

Supp. Fig. S7. Western blot detection of the three marker proteins in tumours and adjacent tissues.

Three frozen OSCC samples (OSCC1, 2 and 3) and two tumour adjacent tissues (Ctl1 and 2) were used to extract total proteins and two blots were prepared.

One blot was used to probe Amphiregulin and the second blot was cut into three pieces, based on protein molecular weight markers, and each was probed with an appropriate antibody as indicated.

In all cases the antibodies detected bands at the expected sizes. Previous works showed that anti-Amphiregulin antibodies usually detects bands at approximately 40, 35, and <20 kDa respectively corresponding to the glycosylated full-length, non-glycosylated full-length and N-terminal-processed protein (Fukuda BBRC 2012, PMID: 22425981; Brown JBC 1998, PMID: 9642297).

Interestingly, our anti-H3 antibody detected H3 at the expected size as well as slower migrating bands above H3 suggesting the occurrence of heavy post-translational modifications of H3, especially in the tumour samples.

Supp. Fig. S7

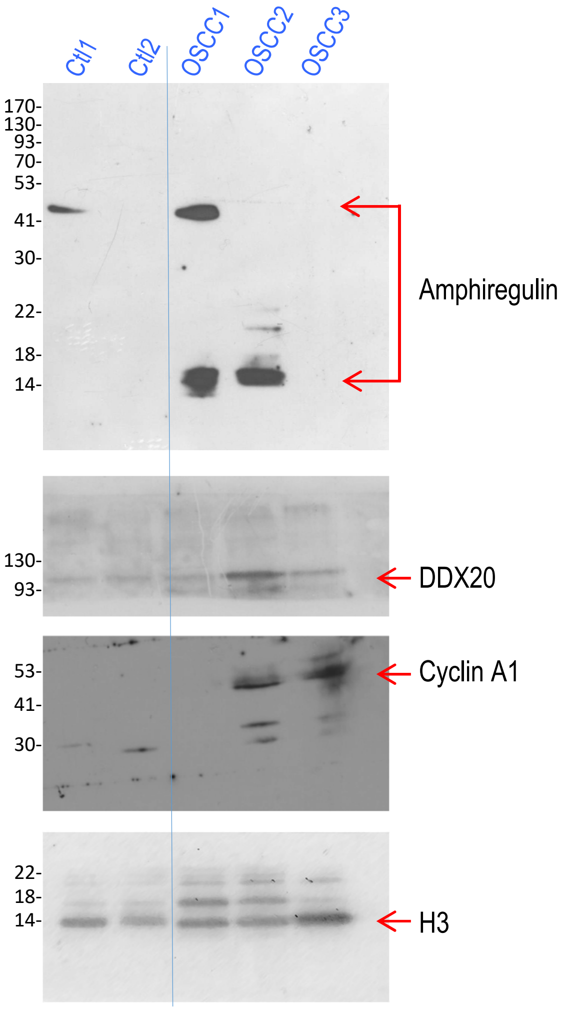

Ctl1
Ctl2
OSCC1
OSCC2
OSCC3
170-
130-
93-
70-
53-
41-
30-
22-
18-
14-
Amphiregulin
130-
93-
DDX20
53-
41-
30-
Cyclin A1
22-
18-
14-
H3

Supp. Fig. S8. Immunohistochemical (IHC) detection of the three marker proteins in our OSCC cohort

IHC detection of *AREG, CCNA1* and *DDX20*, as indicated, showing samples with "high" and "low" labelling at a x400 magnification. The red rectangles correspond to the images shown in Fig 3.

Supp. Fig. S8 x400

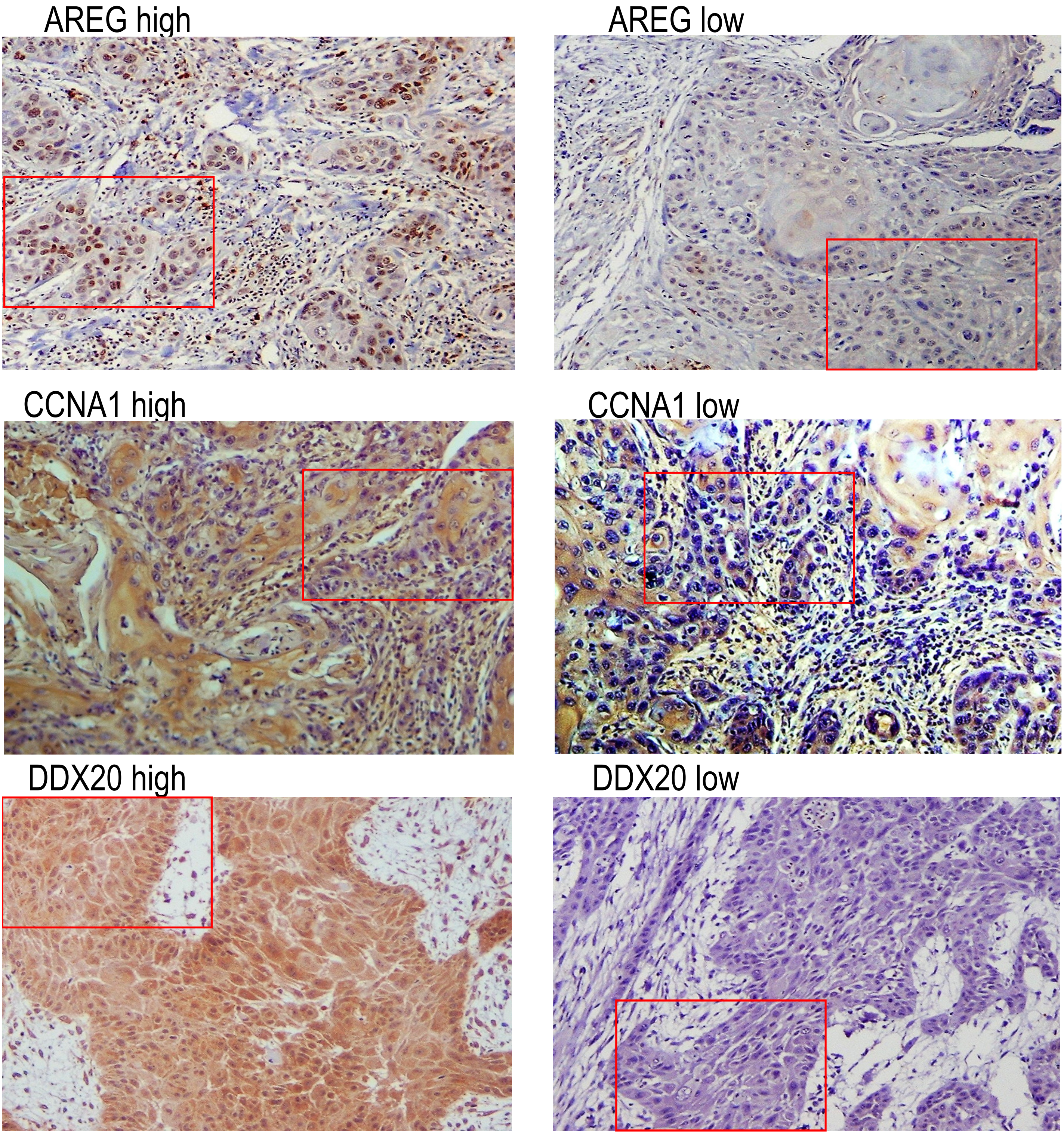

Supp. Fig. S9. Molecular profile of aggressive OSCC tumours visualized by Gene Sets Enrichment Analysis (GSEA) plots. A selection of genesets significantly enriched or depleted are displayed to illustrate the main characteristics of aggressive OSCC tumours.
A and B. GSEA was performed on the transcriptomic signatures of aggressive 3-genes positive versus negative OSCC tumours from the GSE41613 (A) and TCGA-HNSC (B) datasets respectively
C. GSEA plots are also shown for the same genesets with the FaDu cell line expressing CCNA1 (WT) or after experimental downregulation of *CCNA1*.
D. GSEA plots obtained for the same genesets with the transcriptomic signature of HPV negative versus positive tumours from the TCGA-HNSC dataset are also shown.
The details about the genesets are available on the MsigDB website (http://www.gsea-msigdb.org/gsea/msigdb/search.jsp). For detailed enrichment score values and composition of the genesets please refer to supp. Table S6.
The plots are showing significant enrichments of 3-genes positive aggressive tumours (A and B) and of CCNA1 high FaDu cells (C) in the following genesets/signatures:
i/ highly cycling and proliferative cells, embryonic stem cells
ii/ poorly differentiated tumours and epithelial mesenchymal transition (EMT) associated genes
iii/ HRAS, TNFA/NFKB and EGF oncogenic signatures

Supp. Fig. S9

	A GSE41613 3genes pos v neg	B TCGA HNSC 3genes pos v neg	C FaDu cells CCNA1 high v low	D TCGA HNSC HPV neg v pos
BENPORAH PROLIFERATION				
REACTOME CELL CYCLE MITOTIC				
WONG EMBRYONIC STEM CELL CORE				
RHODES UNDIFFERENTIATED CANCER				
SARRIO EPITHELIAL MESENCHYMAL TRANSITION UP				
BILD HRAS ONCOGENIC SIGNATURE				
HALLMARK TNFA SIGNALING VIA NFKB				
NAGASHIMA EGF SIGNALING UP				

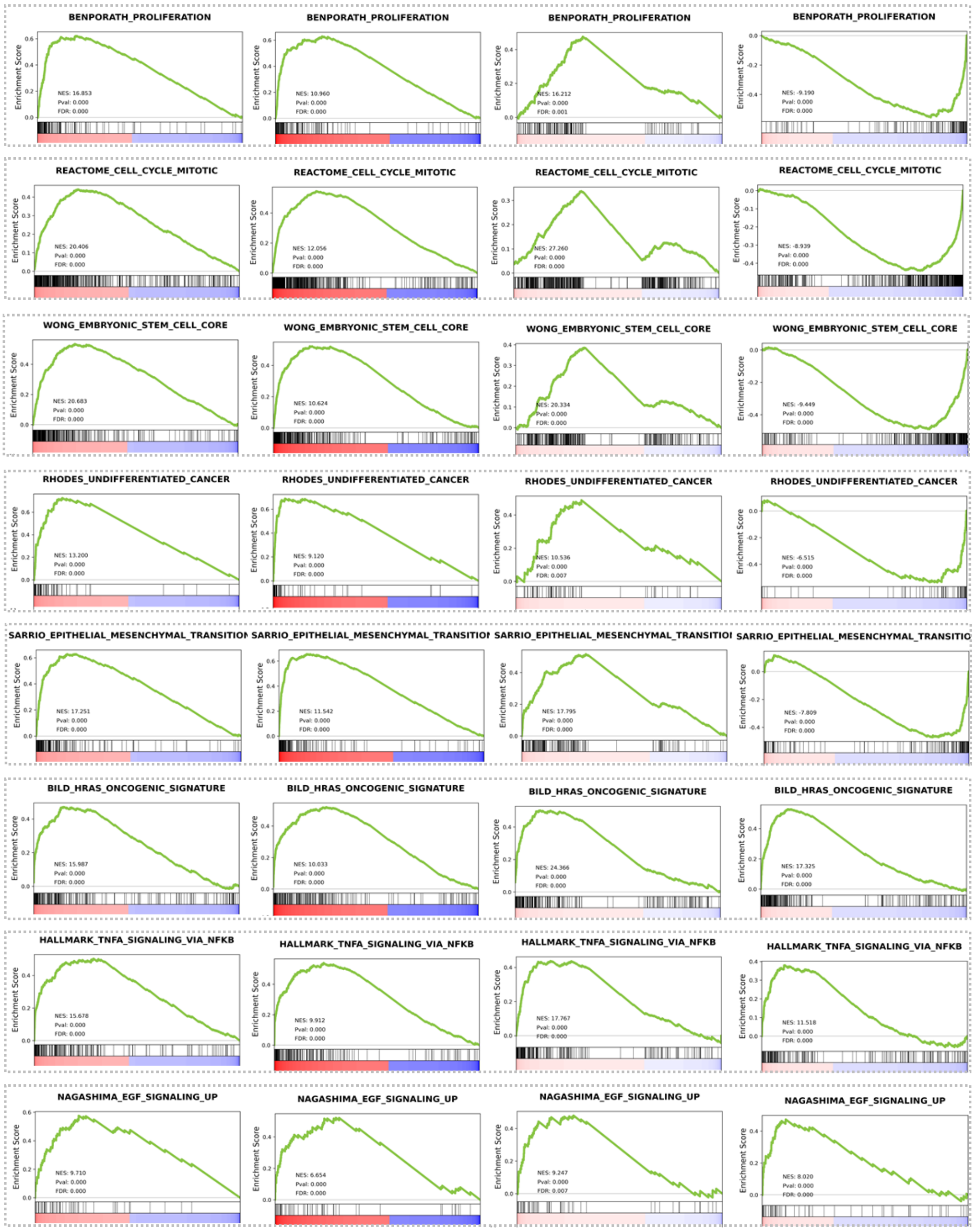

Supp. Fig S10 - Expression of the three genes constituting the 3-genes classifier in normal tissues.
AREG is mainly expressed in the placenta, whereas *CCNA1* and *DDX20* are predominantly expressed in male germ cells.

Supp. Fig. S10

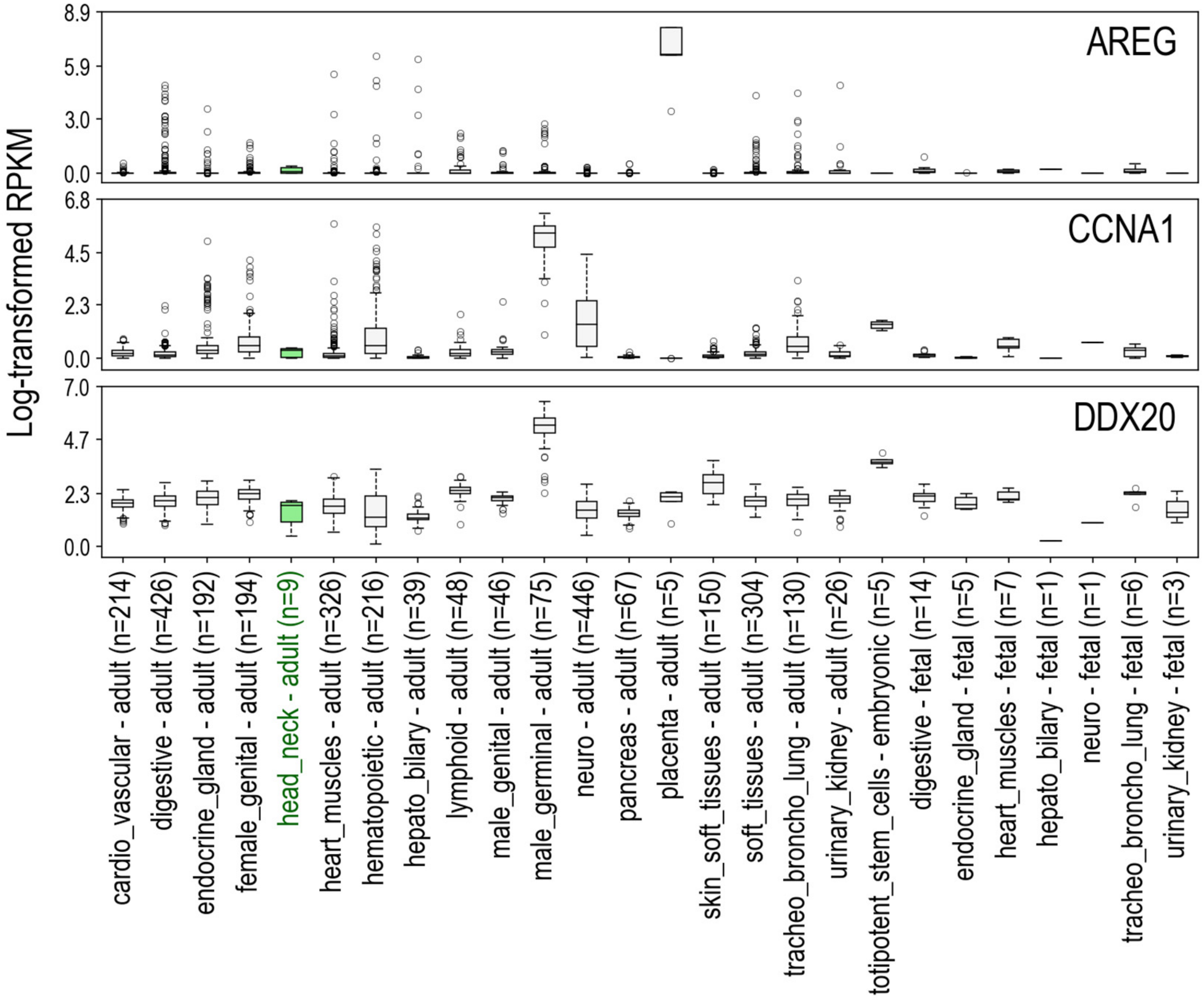
Log-transformed RPKM
AREG
CCNA1
DDX20
8.9
5.9
3.0
0.0
6.8
4.5
2.3
0.0
7.0
4.7
2.3
0.0
cardio_vascular - adult (n=214)
digestive - adult (n=426)
endocrine_gland - adult (n=192)
female_genital - adult (n=194)
head_neck - adult (n=9)
heart_muscles - adult (n=326)
hematopoietic - adult (n=216)
hepato_bilary - adult (n=39)
lymphoid - adult (n=48)
male_genital - adult (n=46)
male_germinal - adult (n=75)
neuro - adult (n=446)
pancreas - adult (n=67)
placenta - adult (n=5)
skin_soft_tissues - adult (n=150)
soft_tissues - adult (n=304)
tracheo_broncho_lung - adult (n=130)
urinary_kidney - adult (n=26)
totipotent_stem_cells - embryonic (n=5)
digestive - fetal (n=14)
endocrine_gland - fetal (n=5)
heart_muscles - fetal (n=7)
hepato_bilary - fetal (n=1)
neuro - fetal (n=1)
tracheo_broncho_lung - fetal (n=6)
urinary_kidney - fetal (n=3)

Legends of Supplemental Tables

Supp. Table S1 – List of 15 genes selected for their association with shorter survival in the OSCC patients from the GSE41613 cohort, a brief description and various identifiers and aliases.

Supp. Table S2 – Association between the 15 selected genes and survival probability of the OSCC patients from the GSE41613 cohort (Affymetrix microarray data) (A) and of the HNSC patients from the TCGA-HNSC cohort (RNAseq data) (B).

Supp. Table S3 – Antibodies used for immunohistochemistry

Supp. Table S4 – Clinical and pathological features of the 66 patients from our cohort of OSCC patients. (A) Detailed clinical and biological parameters and immunohistochemistry results. (B) Descriptive overview of clinical and pathological characteristics of our cohort.

Supp. Table S5 – Multivariate analyses testing the association between 3-genes stratification and prognosis. The tables show, for each of the three cohorts, the results for our 3-genes classifier as well as available clinical and pathological characteristics with the corresponding p-values, hazard ratios (HR) and HR 95% confidence intervals (CI). Significance: *, ** and *** respectively correspond to p-values < 0.05, <0.01 and <0.001. For each table, n corresponds to the number of patients entering the test.

Supp. Table S6 – GSEA enrichment scores (es), normalized enrichment scores (nes), nominal p-values (pval) for a selection of genesets, for each of the transcriptomic analyses considered in Fig 5E and Supp. Fig S9. Information relative to the genesets composition, including the number of genes and their identity is also given.

Supp. Table S7 –Knowledge mining on the potential functional/prognosis impact of our 15 genes in oral squamous cell carcinomas or related cancers.
