# Supplementary material for: The combined detection of Amphiregulin, Cyclin A1 and DDX20/Gemin3 expression predicts aggressive forms of oral squamous cell carcinoma
Source: Br J Cancer. 2021 Jul 21;125(8):1122–34. doi: 10.1038/s41416-021-01491-x (PMC8505643; doi:10.1038/s41416-021-01491-x)
Supplement: Supplementary file 3 — REMARK checklist [file 41416_2021_1491_MOESM3_ESM.pdf]

The REMARK checklist

	Item to be reported	Page no.
**INTRODUCTION**		
1	State the marker examined, the study objectives, and any pre-specified hypotheses.	End of introduction (page 4)
**MATERIALS AND METHODS**		
Patients		
2	Describe the characteristics (e.g., disease stage or co-morbidities) of the study patients, including their source and inclusion and exclusion criteria.	Main Text: Patients and biological samples (page 7) + table S4
3	Describe treatments received and how chosen (e.g., randomized or rule-based).	Main text: Patients and biological samples (page 7) + table S4
Specimen characteristics		
4	Describe type of biological material used (including control samples) and methods of preservation and storage.	Main text: Patients and biological samples (page 8)
Assay methods		
5	Specify the assay method used and provide (or reference) a detailed protocol, including specific reagents or kits used, quality control procedures, reproducibility assessments, quantitation methods, and scoring and reporting protocols. Specify whether and how assays were performed blinded to the study endpoint.	Main text: Analysis of immune-stained samples (page 9) + table S3 (antibodies)<br>Fig 3A (right panel, for scoring)
Study design		
6	State the method of case selection, including whether prospective or retrospective and whether stratification or matching (e.g., by stage of disease or age) was used. Specify the time period from which cases were taken, the end of the follow-up period, and the median follow-up time.	Study design (pages 5-6), Main text: Patients and biological samples (page 7), Supplementary Methods (Data, page 2)
7	Precisely define all clinical endpoints examined.	Study design (pages 5-6)<br>Main text: Patients and biological samples (page 7)
8	List all candidate variables initially examined or considered for inclusion in models.	Study design (pages 5-6)
9	Give rationale for sample size; if the study was designed to detect a specified effect size, give the target power and effect size.	Supplemental Method (Sample size, page 2)
Statistical analysis methods		
10	Specify all statistical methods, including details of any variable selection procedures and other model-building issues, how model assumptions were verified, and how missing data were handled.	Supp. Methods: Mining publicly available transcriptomic data (pages 3-5)
11	Clarify how marker values were handled in the analyses; if relevant, describe methods used for cutpoint determination.	Main text: Immunohistochemistry assay (page 8) and Analysis of immuno-stained samples (page 9)
**RESULTS**		
Data		
12	Describe the flow of patients through the study, including the number of patients included in each stage of the analysis (a diagram may be helpful) and reasons for dropout. Specifically, both overall and for each subgroup extensively examined report the numbers of patients and the number of events.	Fig 1<br>Table S4A and B
13	Report distributions of basic demographic characteristics (at least age and sex), standard (disease-specific) prognostic variables, and tumor marker, including numbers of missing values.	Table S4A and B
Analysis and presentation		
14	Show the relation of the marker to standard prognostic variables.	Fig 2 BCD

Source: McShane LM, Altman DG, Sauerbrei W, Taube SE, Gion M, Clark GM: Reporting recommendations for tumor marker prognostic studies (REMARK). *J Natl Cancer Inst* 2005; 97: 1180-1184.

15	Present univariable analyses showing the relation between the marker and outcome, with the estimated effect (e.g., hazard ratio and survival probability). Preferably provide similar analyses for all other variables being analyzed. For the effect of a tumor marker on a time-to-event outcome, a Kaplan-Meier plot is recommended.	Fig1, 2 and 3BC<br>Supp. Fig S1, S2, S3, S4, S5, S6
16	For key multivariable analyses, report estimated effects (e.g., hazard ratio) with confidence intervals for the marker and, at least for the final model, all other variables in the model.	Table S5
17	Among reported results, provide estimated effects with confidence intervals from an analysis in which the marker and standard prognostic variables are included, regardless of their statistical significance.	Table S5
18	If done, report results of further investigations, such as checking assumptions, sensitivity analyses, and internal validation.	Internal robustness criteria described in Supplementary Methods (page 4)
**DISCUSSION**		
19	Interpret the results in the context of the pre-specified hypotheses and other relevant studies; include a discussion of limitations of the study.	Discussion (pages 16-18)
20	Discuss implications for future research and clinical value.	End of discussion (page 18)

Source: McShane LM, Altman DG, Sauerbrei W, Taube SE, Gion M, Clark GM: Reporting recommendations for tumor marker prognostic studies (REMARK). *J Natl Cancer Inst* 2005; 97: 1180-1184.
