# Supplementary material for: The combined detection of Amphiregulin, Cyclin A1 and DDX20/Gemin3 expression predicts aggressive forms of oral squamous cell carcinoma
Source: Br J Cancer. 2021 Jul 21;125(8):1122–34. doi: 10.1038/s41416-021-01491-x (PMC8505643; doi:10.1038/s41416-021-01491-x)
Supplement: Supplementary file 4 — authors list agreement [file 41416_2021_1491_MOESM4_ESM.docx]

**From:**pouyan aminishakib <pouyanshakib@yahoo.com>

**Subject: Re: URGENT Fwd: MD-2021-3104R1 Initial Quality Check of your BJC Manuscript**

**Date:**18 June 2021 at 10:53:14 CEST

**To:**Saadi Khochbin <saadi.khochbin@univ-grenoble-alpes.fr>, Sophie Rousseaux <sophie.rousseaux@univ-grenoble-alpes.fr>

Dear Sophie,

I would like to express my agreement with the authors' list of the manuscript entitled "The combined detection of Amphiregulin, Cyclin A1 and DDX20/Gemin3

expression predicts aggressive forms of Oral Squamous Cell Carcinoma".

Thank you for your all efforts.

With warm regards.

Pouyan

Pouyan Aminishakib, DDS, MSc

Associate Professor of Oral and Maxillofacial Pathology Department,

Vice-Dean for International Affairs of School of Dentistry,

Tehran University of Medical Sciences

1439955991; Tehran, Iran

Tel: ++98 21 88351163

Fax: ++98 21 88015800

Email: aminishakib@tums.ac.ir

Web: www.tums.ac.ir

**From:**TAO WANG <tao.wang@univ-grenoble-alpes.fr>

**Subject: Re: URGENT Fwd: MD-2021-3104R1 Initial Quality Check of your BJC Manuscript**

**Date:**18 June 2021 at 10:10:06 CEST

**To:**SOPHIE ROUSSEAUX <sophie.rousseaux@univ-grenoble-alpes.fr>

**Cc:**Ekaterina Flin <ekaterina.flin@univ-grenoble-alpes.fr>, Samira Derakhshan <samderakhshan.den@gmail.com>, afsaneh goudarzi <afsaneh.goudarzi@sbmu.ac.ir>, ANNE LAURE VITTE <anne-laure.vitte@univ-grenoble-alpes.fr>, florent chuffart <florent.chuffart@univ-grenoble-alpes.fr>, Saadi Khochbin <saadi.khochbin@univ-grenoble-alpes.fr>, pouyan aminishakib <aminishakib@tums.ac.ir>

Dear Sophie,

I'm sending this email to confirm that I agree with the authors list of the manuscript.

Thank you again for involving me in this wonderful project.

Best wishes,

Tao

**From:**ANNE LAURE VITTE <anne-laure.vitte@univ-grenoble-alpes.fr>

**Subject: Re: URGENT Fwd: MD-2021-3104R1 Initial Quality Check of your BJC Manuscript**

**Date:**18 June 2021 at 10:54:46 CEST

**To:**Sophie Rousseaux <sophie.rousseaux@univ-grenoble-alpes.fr>

**Cc:**Ekaterina Flin <ekaterina.flin@univ-grenoble-alpes.fr>, Samira Derakhshan <samderakhshan.den@gmail.com>, afsaneh goudarzi <afsaneh.goudarzi@sbmu.ac.ir>, TAO WANG <tao.wang@univ-grenoble-alpes.fr>, Florent Chuffart <florent.chuffart@univ-grenoble-alpes.fr>, Saadi Khochbin <saadi.khochbin@univ-grenoble-alpes.fr>, pouyan aminishakib <aminishakib@tums.ac.ir>

Dear Sophie ,
I confirm that I agree with the authors list for the manuscript.  
Thank you 
Anne Laure 
----- Mail d’origine -----
De: Sophie Rousseaux <sophie.rousseaux@univ-grenoble-alpes.fr>
À: Ekaterina Flin <ekaterina.flin@univ-grenoble-alpes.fr>, Samira Derakhshan <samderakhshan.den@gmail.com>, afsaneh goudarzi <afsaneh.goudarzi@sbmu.ac.ir>, TAO WANG <tao.wang@univ-grenoble-alpes.fr>, ANNE LAURE VITTE <anne-laure.vitte@univ-grenoble-alpes.fr>, Florent Chuffart <florent.chuffart@univ-grenoble-alpes.fr>, Saadi Khochbin <saadi.khochbin@univ-grenoble-alpes.fr>, pouyan aminishakib <aminishakib@tums.ac.ir>
Envoyé: Thu, 17 Jun 2021 16:57:13 +0200 (CEST)
Objet: URGENT Fwd: MD-2021-3104R1 Initial Quality Check of your BJC Manuscript

**From:**afsaneh.goudarzi@sbmu.ac.ir

**Subject: Re: URGENT Fwd: MD-2021-3104R1 Initial Quality Check of your BJC Manuscript**

**Date:**17 June 2021 at 19:07:59 CEST

**To:**"Saadi Khochbin sophie.rousseaux@univ-grenoble-alpes.fr" <saadi.khochbin@univ-grenoble-alpes.fr>, ekaterina.flin@univ-grenoble-alpes.fr, aminishakib@tums.ac.ir, samderakhshan.den@gmail.com, anne-laure.vitte@univ-grenoble-alpes.fr, florent.chuffart@univ-grenoble-alpes.fr, tao.wang@univ-grenoble-alpes.fr

**Cc:**"Sophie Rousseaux" <sophie.rousseaux@univ-grenoble-alpes.fr>, "Ekaterina Flin" <ekaterina.flin@univ-grenoble-alpes.fr>, "Samira Derakhshan" <samderakhshan.den@gmail.com>, afsaneh.goudarzi@sbmu.ac.ir, "TAO WANG" <tao.wang@univ-grenoble-alpes.fr>, "ANNE LAURE VITTE" <anne-laure.vitte@univ-grenoble-alpes.fr>, "Florent Chuffart" <florent.chuffart@univ-grenoble-alpes.fr>, "pouyan aminishakib" <aminishakib@tums.ac.ir>

Dear Sophie,

I am sending this email to confirm that I agree with the authors list of this manuscript.

With best wishes,

Afsaneh Goudarzi

**From:**Saadi Khochbin <saadi.khochbin@univ-grenoble-alpes.fr>

**Subject: Re: URGENT Fwd: MD-2021-3104R1 Initial Quality Check of your BJC Manuscript**

**Date:**17 June 2021 at 18:15:08 CEST

**To:**Sophie Rousseaux <sophie.rousseaux@univ-grenoble-alpes.fr>

**Cc:**Ekaterina Flin <ekaterina.flin@univ-grenoble-alpes.fr>, Samira Derakhshan <samderakhshan.den@gmail.com>, afsaneh.goudarzi@sbmu.ac.ir, TAO WANG <tao.wang@univ-grenoble-alpes.fr>, ANNE LAURE VITTE <anne-laure.vitte@univ-grenoble-alpes.fr>, Florent Chuffart <florent.chuffart@univ-grenoble-alpes.fr>, pouyan aminishakib <aminishakib@tums.ac.ir>

Dear Sophie,

I agree with the authors list of this manuscript.

With best wishes

Saadi Khochbin

**From:**Florent Chuffart <florent.chuffart@univ-grenoble-alpes.fr>

**Subject: Re: URGENT Fwd: MD-2021-3104R1 Initial Quality Check of your BJC Manuscript**

**Date:**17 June 2021 at 16:59:10 CEST

**To:**Sophie Rousseaux <sophie.rousseaux@univ-grenoble-alpes.fr>

Dear Sophie, 

I confirm you that I'm agree with the new authors list of our paper.

Best Regards, 


—
Florent Chuffart 
IAB - INSERM (U1209) - CNRS (UMR5309) 
Site Santé - Allée des Alpes 
38700 La Tronche - France
florent.chuffart@univ-grenoble-alpes.fr
tel: +33 (0)4 76 54 95 82
fax: +33 (0)4 76 54 94 25

**From:**Samira Derakhshan <samderakhshan.den@gmail.com>

**Subject: Re: URGENT Fwd: MD-2021-3104R1 Initial Quality Check of your BJC Manuscript**

**Date:**18 June 2021 at 07:50:14 CEST

**To:**Sophie Rousseaux <sophie.rousseaux@univ-grenoble-alpes.fr>

**Cc:**Ekaterina Flin <ekaterina.flin@univ-grenoble-alpes.fr>, afsaneh.goudarzi@sbmu.ac.ir, TAO WANG <tao.wang@univ-grenoble-alpes.fr>, ANNE LAURE VITTE <anne-laure.vitte@univ-grenoble-alpes.fr>, Florent Chuffart <florent.chuffart@univ-grenoble-alpes.fr>, Saadi Khochbin <saadi.khochbin@univ-grenoble-alpes.fr>, pouyan aminishakib <aminishakib@tums.ac.ir>

Dear Sophie

I am sending this email to confirm that I agree with the authors list of the manuscript.

Thank you very much

With best wishes

Samira

**From:**Ekaterina Flin <ekaterina.flin@univ-grenoble-alpes.fr>

**Subject: Re: URGENT Fwd: MD-2021-3104R1 Initial Quality Check of your BJC Manuscript**

**Date:**17 June 2021 at 17:03:01 CEST

**To:**Sophie Rousseaux <sophie.rousseaux@univ-grenoble-alpes.fr>

Dear Sophie,

I totally agree with the updated version of the paper.

Best wishes,

Katia

On 17 Jun 2021, at 16:57, Sophie Rousseaux <sophie.rousseaux@univ-grenoble-alpes.fr> wrote:

Dear all,

I hope you are all well

BJC is asking each of you to confirm that you agree with the authors list of our paper.
Indeed, in the initial submission I forgot to add Tao, who contributed with Anne-Laure to the CCNA1 knock down experiments.
Please find a copy of the authors list below and the merged pdf of the last submission

Would you please send me your agreement with the authors list by repying to this email at your earliest convenience?

As requested by the journal, I will compile all your answers in a single document which I will upload on he site.

I wish you a nice evening

With best regards

Sophie

The combined detection of Amphiregulin, Cyclin A1 and DDX20/Gemin3 expression predicts aggressive forms of Oral Squamous Cell Carcinoma
Ekaterina Bourova-Flin1, Samira Derakhshan2, Afsaneh Goudarzi3, Tao Wang1, Anne-Laure Vitte1, Florent Chuffart 1, Saadi Khochbin1, Sophie Rousseaux1*, Pouyan Aminishakib2*

*Corresponding author Email: aminishakib@tums.ac.ir (PA, ORCID: 0000-0002-2185- 0050); sophie.rousseaux@univ-grenoble-alpes.fr (S.R., ORCID: 0000-0001-5246-5350)
Affiliations:
1 CNRS UMR 5309/INSERM U1209/University Grenoble-Alpes/Institute for Advanced Biosciences, La Tronche, France.
2 Oral and Maxillofacial Pathology Department, School of Dentistry, Tehran University of Medical Sciences, Tehran, Iran.
3 Department of Clinical Biochemistry, School of Medicine, Shahid Beheshti University of Medical Sciences, Tehran, Iran.

Begin forwarded message:

From: bjc@springernature.com
Subject: MD-2021-3104R1 Initial Quality Check of your BJC Manuscript
Date: 17 June 2021 at 15:17:17 CEST
To: sophie.rousseaux@univ-grenoble-alpes.fr
Reply-To: bjc@springernature.com

17th Jun 2021

Dear Professor Rousseaux,

After checking the manuscript that you submitted to British Journal of Cancer, it has come to our attention that the following item(s) need(s) to be addressed before we can proceed:

1. Please make sure all figures are cited in numerical order

2. It has come to our attention that your most recent author list differs from the one in your original submission. 

Please request agreement from all authors including additions and deletions, these can be collected in the following way:

Email your co-authors with the change, and ask them to reply to your email confirming that they agree to these changes. Once you have collected these replies, please combine all of the co-authors’ email responses in one document and upload this file to your submission.


We have returned your manuscript to the Author Approval Folder, where you can access it using the following link: https://mts-bjcancer.nature.com/cgi-bin/main.plex?el=A6BV3YIL4C1BXFY5F3A9ftdJQbAmIkotJC50Z4gRqrVnwZ

(Press/Click on the above link to be automatically sent to the web page.) 

Once you have made the requested changes to your submission please submit your manuscript as you did before. If you have any questions please contact the BJC Editorial Office at bjc@springernature.com, quoting manuscript number MD-2021-3104R1. Thank you.

Yours sincerely,

Lauren Walsh
1
British Journal of Cancer


*Our flexible approach during the COVID-19 pandemic*
If you need more time at any stage of the peer-review process, please do let us know. While our systems will continue to remind you of the original timelines, we aim to be as flexible as possible during the current pandemic.

This email has been sent through the NPG Manuscript Tracking System NY-610A-NPG&MTS

Confidentiality Statement:

This e-mail is confidential and subject to copyright. Any unauthorised use or disclosure of its contents is prohibited. If you have received this email in error please notify our Manuscript Tracking System Helpdesk team at http://platformsupport.nature.com .

Details of the confidentiality and pre-publicity policy may be found here http://www.nature.com/authors/policies/confidentiality.html

Privacy Policy | Update Profile

<bourova_bjc_main_merged.pdf>
